# Evaluation of finite element modeling methods for predicting compression screw failure in a custom pelvic implant

**DOI:** 10.3389/fbioe.2024.1420870

**Published:** 2024-08-20

**Authors:** Yuhui Zhu, Ata Babazadeh-Naseri, Matthew R. W. Brake, John E. Akin, Geng Li, Valerae O. Lewis, Benjamin J. Fregly

**Affiliations:** ^1^ Department of Mechanical Engineering, Rice University, Houston, TX, United States; ^2^ Department of Orthopedic Oncology, University of Texas MD Anderson Cancer Center, Houston, TX, United States

**Keywords:** pelvic implant, custom prosthesis design, finite element analysis, fixation durability, fixation failure, compression screws

## Abstract

**Introduction:** Three-dimensional (3D)-printed custom pelvic implants have become a clinically viable option for patients undergoing pelvic cancer surgery with resection of the hip joint. However, increased clinical utilization has also necessitated improved implant durability, especially with regard to the compression screws used to secure the implant to remaining pelvic bone. This study evaluated six different finite element (FE) screw modeling methods for predicting compression screw pullout and fatigue failure in a custom pelvic implant secured to bone using nine compression screws.

**Methods:** Three modeling methods (tied constraints (TIE), bolt load with constant force (BL-CF), and bolt load with constant length (BL-CL)) generated screw axial forces using functionality built into Abaqus FE software; while the remaining three modeling methods (isotropic pseudo-thermal field (ISO), orthotropic pseudo-thermal field (ORT), and equal-and-opposite force field (FOR)) generated screw axial forces using iterative physics-based relationships that can be implemented in any FE software. The ability of all six modeling methods to match specified screw pretension forces and predict screw pullout and fatigue failure was evaluated using an FE model of a custom pelvic implant with total hip replacement. The applied hip contact forces in the FE model were estimated at two locations in a gait cycle. For each of the nine screws in the custom implant FE model, likelihood of screw pullout failure was predicted using maximum screw axial force, while likelihood of screw fatigue failure was predicted using maximum von Mises stress.

**Results:** The three iterative physics-based modeling methods and the non-iterative Abaqus BL-CL method produced nearly identical predictions for likelihood of screw pullout and fatigue failure, while the other two built-in Abaqus modeling methods yielded vastly different predictions. However, the Abaqus BL-CL method required the least computation time, largely because an iterative process was not needed to induce specified screw pretension forces. Of the three iterative methods, FOR required the fewest iterations and thus the least computation time.

**Discussion:** These findings suggest that the BL-CL screw modeling method is the best option when Abaqus is used for predicting screw pullout and fatigue failure in custom pelvis prostheses, while the iterative physics-based FOR method is the best option if FE software other than Abaqus is used.

## 1 Introduction

Pelvic sarcoma is a malignant tumor that affects the bones and muscles of the pelvis. Its treatment typically involves complete surgical resection of the tumor, which often leaves a sizeable bone defect in the pelvis. In some cases, surgeons use a three-dimensional (3D)-printed custom pelvic implant to reconstruct the pelvic bony anatomy. The custom implant is typically designed to fill the hole left by the resected tumor using a shape mirrored from the healthy contralateral pelvis, as determined from the patient’s pre-surgery imaging data. This design approach allows surgeons to match the desired surgical resection planes precisely and recreate the original complex bony geometry of the pelvis. Compared to tumor resection without custom implant reconstruction, use of 3D-printed custom pelvic implants reduces post-operative recovery time and produces more normal post-operative walking function (Liang et al., 2017; Vega et al., 2022). These promising outcomes, coupled with the ease of custom implant design and fabrication, have led to the growing use of these implants in recent years (Liang et al., 2017; Angelini et al., 2019; Chen et al., 2020; Wang et al., 2022; Xu et al., 2022).

With the increased clinical use of custom pelvic implants has come an increased emphasis on evaluating and improving their durability. While the reliability of custom pelvic implants has improved over time (Ji and Guo, 2019), recent studies have reported a significant number of screw mechanical failures, such as loosening due to pullout failure (Ozaki et al., 2002; Sun et al., 2011; Broekhuis et al., 2022; Zhu et al., 2023) and breakage due to fatigue failure (Ozaki et al., 2002; Shao et al., 2015; Ji et al., 2020; Zhu et al., 2023). The high occurrences of screw failures may be attributable to insufficient engineering evaluation in the typical design process of custom pelvic implants. Currently, the typical design process begins with using the patient’s imaging data to create a surgical resection plan that removes the tumor with clear margins. Thereafter, a custom implant design is developed that recapitulates the resected bony anatomy by mirroring the contralesional pelvis with the surgical resection planes. Lastly, the locations and trajectories of multiple fixation screws are specified to secure the custom implant to the pelvis, and the corresponding screw holes are added to finalize the custom implant design. In this design process, the operating surgeon must make important custom implant design decisions based on subjective clinical judgment. Especially when the underlying biomechanical relationships are not well understood, the surgeon may need to explore new design concepts by following a trial-and-error process. For example, in an effort to improve fixation stability, Ji et al. (2020) experimented with using different orientations for the main fixation screws on custom implant designs over a 3-year period (Ji et al., 2020), while Wang et al. (2019) explored various screw layouts and fixation techniques on a series of implant designs (Wang et al., 2019). While both studies reported satisfactory early outcomes, limited biomechanical analyses were performed to evaluate these new fixation strategies, making it difficult to identify generalized design principles that reduce the incidence of screw mechanical failures. Ideally, engineering evaluation would be incorporated into the design process to assess fixation durability proactively before clinical failures occur.

Recent studies have begun to explore the use of finite element (FE) modeling methods to improve the durability of fixation screws used in custom pelvic implants (Zhou et al., 2013; 2022; Wang et al., 2015; Iqbal et al., 2017; Dong et al., 2018; 2019; Park et al., 2019; Maslov et al., 2021; Guo et al., 2023; Soloviev et al., 2023; Zhu et al., 2023). Maslov et al. (2021) demonstrated that the magnitude of screw pretension forces used in a pelvic FE model can alter the simulated stress distribution experienced by the custom implant and the remaining pelvic bone (Maslov et al., 2021). The study further concluded that fixation screws should be tightened enough to encourage bone regeneration but not excessively to cause local bone destruction. Zhu et al. (2023) used a post-operative hemipelvis FE model to demonstrate that different ways of modeling bone-implant interaction, indicative of different stages of osseointegration, can have a substantial effect on predicted fixation durability of locking screws (Zhu et al., 2023). The study concluded that osseointegration is important for the stability of the reconstructed pelvis. Both studies highlighted how different FE modeling decisions can have a large effect on predicted screw durability. When making modeling decisions, it is critical that relevant physiological relationships are represented within the model assembly to maximize the fidelity of the simulation results.

One aspect that has often been overlooked in post-surgery pelvic FE models is the technique for modeling screw fixation. Implant fixation screws can be generally categorized into two groups: locking screws and compression screws. Locking screws use a threaded head and a matching threaded screw hole. As the screw is tightened, the screw head becomes “locked” to the threaded screw hole and is hard to separate from the implant. In contrast, compression screws use a conventional non-locking screw to compress the implant against the bone. Unlike locking screws, compression screws remain in contact with the implant through a pretension force within the screw core converted from the torque applied to tighten the screw. Many FE studies of custom pelvic implants have used tie constraints between the screw head and the implant, bonding the two components together (Iqbal et al., 2017; Dong et al., 2018; Park et al., 2019; Zhou et al., 2022; Guo et al., 2023; Zhu et al., 2023). However, this screw modeling method is not appropriate for compression screws, where the screw head is simply compressing the implant. When modeling a compression screw, over-constraining the model by tying the screw head and implant together might lead implant designers to reach incorrect conclusions about fixation durability. A method for modeling compression screws is crucial for accurately evaluating the fixation durability of custom implants that are secured using such screws.

In the available literature, various studies have proposed different screw models where a pretension step is implemented before the simulation step to model compression screws. In the first step (*i.e.*, pretension step), a pretension force is realized within the screw core to compress the screw against the implant without the application of any external loads, resembling the tightening of the screw. In the second step (*i.e.*, simulation step), the external loads are applied to the FE model to simulate the stress distribution within the screw or its surrounding structures. Different screw modeling methods differ in how the pretension force is realized in the pretension step. Some recent pelvic FE studies used an Abaqus built-in loading method called “bolt load” to realize the pretension force in each compression screw (Dong et al., 2018; Maslov et al., 2021; Soloviev et al., 2023). However, little information is available to understand the theory behind the built-in bolt load method. Other studies utilized physics-based methods to realize the pretension force. Alkan et al. (2004) imposed a pseudo thermal field to the unthreaded shank of the screw in a dental implant system, thereby producing a pseudo temperature change that in turn produced a thermal contraction, creating a pretension force within the screw that compressed two dental implant components together (Alkan et al., 2004). However, this screw model had not been implemented to analyze custom pelvic implants utilizing multiple fixation screws. Alternatively, when simulating a bolted joint, Balaji et al. (2020) used a set of two equal and opposite compressive forces along the axial direction of a bolt to realize the pretension force (Balaji et al., 2020). The compressive forces were applied to the contacting surfaces of the bolt and the nut, respectively, and the contacting surfaces are constrained to relative movements only in the axial direction. However, this method has not been adapted to model compression screws, nor has it been investigated for analysis of custom pelvic implants. While these screw modeling methods appear promising for simulating compression screws in a post-surgery pelvis FE model, it is unknown how they compare when used to assess pullout and fatigue failure of compression screws used for pelvic reconstruction. To the authors’ knowledge, no study has surveyed and compared the potential methods of modeling compression screws in a pelvis FE model, and consequently, the most appropriate method for modeling compression screws when analyzing fixation durability has yet to be determined.

This study investigated how different FE methods for modeling compression screw fixation in a custom pelvic implant affected predictions of screw pullout and fatigue failures under gait loading conditions. An FE model was constructed of the bone-implant-screw system for a specific pelvic sarcoma patient using a combination of walking, imaging, and custom implant geometry data collected from the patient. Six distinct compression screw modeling methods were implemented in the FE model, and pullout and fatigue failure predictions produced by each method were compared. The study demonstrates the importance of selecting a physiological screw model and identifies reliable compression screw modeling methods that could be used in future fixation durability analyses of custom pelvic implants.

## 2 Materials and methods

### 2.1 Experimental data collection

Surgical plan, custom pelvic implant, pre- and post-surgery computed tomography (CT), and walking data were collected from a 46-year-old male subject (height: 1.73 m, weight: 85 kg) diagnosed with a pelvic sarcoma in the acetabular region of the right hemipelvis. The subject underwent Enneking Type-II resection followed by endo-prosthetic reconstruction using a commercial custom pelvic implant (Onkos Surgical, United States) with total hip replacement. Pelvic tumor resection and subsequent pelvic reconstruction were performed during a single surgical session at MD Anderson Cancer Center in Houston, TX. Institutional review board (IRB) approvals were obtained from the University of Texas Health Science Center, MD Anderson Cancer Center, and Rice University for collection and analysis of walking, imaging, surgical, and custom pelvic prosthesis data, and subject provided written informed consent for all collected data.

**FIGURE 1:**
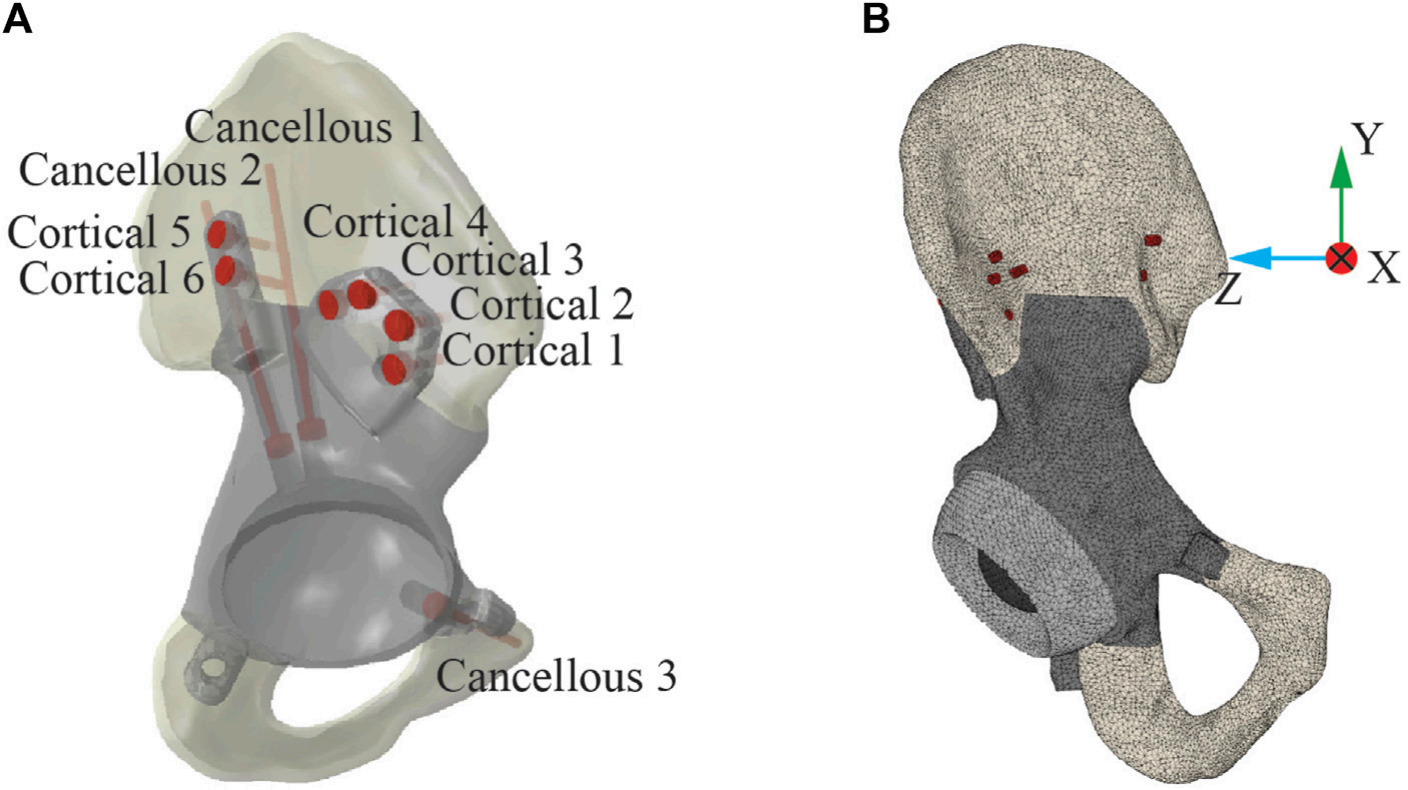
Post-operative geometric model of the ipsilesional hemipelvis with custom pelvic implant. **(A)** The layout of the nine screws used to fix the implant to the remaining bone. Three screws were “cancellous” screws, which were inserted from the acetabular cup of the implant into the cancellous region of the remaining ilium bone. The remaining six screws were “cortical” screws, which were inserted medially through the implant extracortical flanges and drilled through two cortical layers with a layer of trabecular bone between them. **(B)** The post-operative FE model of the ipsilesional hemipelvis with custom pelvic implant.

After multi-planar pelvic bone resection was planned to remove the tumor with clear margins, the subject’s custom pelvic implant was designed to recapitulate the anatomical shape of his contralesional hemipelvis. The implant was made of 3D-printed biomedical-grade titanium alloy Ti-6Al-4V (Ti64) and secured to remaining bone through nine commercially available Ti64 compression screws (inner diameter: 3.0 mm, outer diameter: 6.5 mm, pitch: 2.75 mm; MicroPort Orthopedics, United States). The location and trajectory of the screws were planned by the operating surgeon and incorporated into the subject’s custom pelvic implant design. Three cancellous bone screws were inserted through the implant’s acetabular cup – two into the ilium and one into the superior pubic ramus, while six bi-cortical screws were inserted through two extracortical flanges into the ilium – four through the anterior flange and two through the posterior flange (Figure 1A).

Prior to surgery and following plateau in recovery after surgery, the subject participated in an experimental walking data collection session at the University of Texas Health Science Center. During each session, the subject completed a static standing trial and a treadmill walking trial at his self-selected speed (1.0 m/s before surgery and 0.5 m/s after surgery). Experimental data collected during these trials included full-body retroreflective marker motion data from a video motion capture system (Qualisys AB, Gothenburg, Sweden), ground reaction data from a split-belt instrumented treadmill with belts tied (Bertec Corporation, Columbus, OH, United States), and wireless electromyography (EMG) data from 16 muscles per leg (Cometa, Bareggio, Italy). These data were used to construct a post-operative musculoskeletal model of the subject (see Section 2.2 below), which in turn was used to estimate the subject’s post-operative ipsilesional hip joint contact forces during gait. These contact forces were incorporated into subsequent FE analyses that evaluated custom pelvic implant screw durability using different FE modeling methods (see Section 2.3 below).

### 2.2 Personalized musculoskeletal model

**FIGURE 2:**
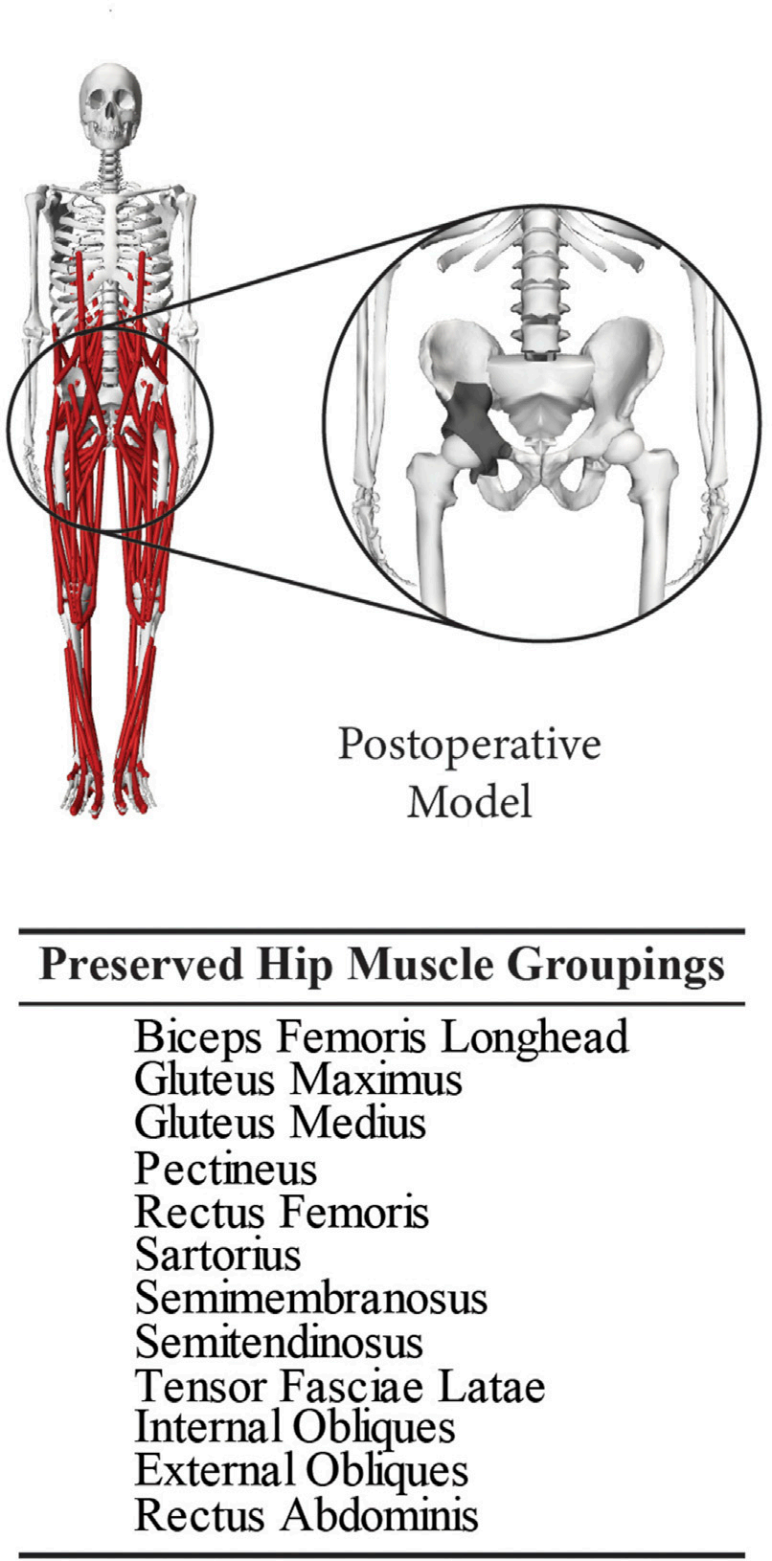
Post-operative personalized OpenSim musculoskeletal model built using the subject’s reconstructed pelvis geometry with surgically resected muscles removed. This model was used to calculate post-surgery hip joint contact forces during a typical gait cycle performed by the subject.

Post-surgery hip joint contact forces during gait were estimated using a personalized musculoskeletal model of the subject. All musculoskeletal modeling work was performed using the OpenSim musculoskeletal modeling software (Delp et al., 2007; Seth et al., 2018) and a published generic OpenSim walking model (Rajagopal et al., 2016). In a previous study, this generic model was transformed into a pre-surgery personalized model of the same subject as in the present study using the subject’s pre-surgery walking and CT scan data (Li et al., 2022). The transformation process involved model scaling, replacing the scaled generic pelvis with the subject’s personalized pelvis geometry, and personalizing the optimal muscle fiber length and tendon slack length values of the model’s Hill-type lower limb muscle-tendon actuators using an EMG-driven modeling method (Ao et al., 2022; Li et al., 2022; 2024). For the present study, the subject’s pre-surgery personalized model was transformed into a post-surgery personalized model using the subject’s post-surgery walking data, custom pelvic implant geometry obtained from the manufacturer, and recorded surgical decisions (Figure 2). The transformation process involved incorporating the subject’s custom pelvic implant geometry into his pre-surgery personalized pelvis geometry, removing the same muscles from the operated side of the model as were removed during surgery, and adjusting the location of the hip joint center on the operated side by a small amount to be consistent with custom implant.

**FIGURE 3:**
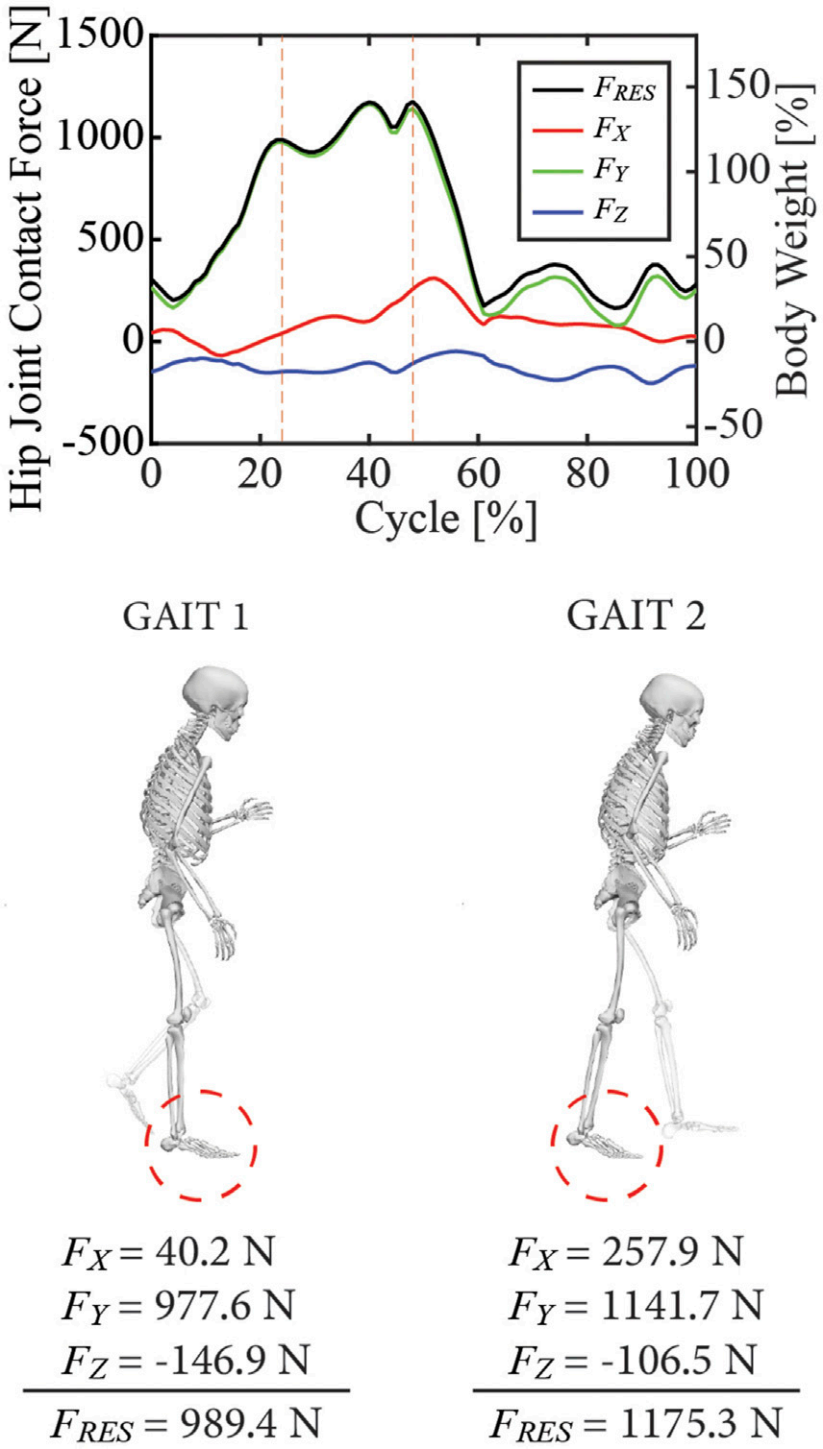
Visual depiction of the two load cases used in the finite element simulations. The locations of the first and third peaks in estimated hip joint contact force, corresponding to foot-flat (named Gait 1) and heel-off (names Gait 2), were selected for analysis. 0% of the gait cycle corresponds to heel-strike of the ipsilesional foot. The X, Y, and Z components of each load case are presented in the pelvic coordinate system, where X, Y, and Z represent anterior-posterior, superior-inferior, and medial-lateral directions, respectively.

Using this post-surgery personalized OpenSim model and data from one representative post-surgery walking cycle, we performed a sequence of four standard OpenSim analyses to estimate hip joint contact forces on the operated side over one gait cycle. First, an Inverse Kinematics analysis was performed to calculate lower limb joint motions. Second, an Inverse Dynamics analysis was performed to calculate the associated lower limb joint moments. Third, a Static Optimization that minimized the sum of squares of muscle activations was performed to estimate leg muscle forces. Fourth, a Joint Reaction Analysis was performed to estimate ipsilesional hip joint contact forces produced by the estimated hip muscle forces. Two peaks of the predicted ipsilesional hip joint contact force, named Gait 1 (corresponding to foot-flat) and Gait 2 (corresponding to heel-off), were selected as the two load cases to be used in the subsequent FE analyses (Figure 3).

### 2.3 Pelvis-implant geometric model

Pre-surgery and post-surgery CT scan data of the subject’s pelvis were used to construct a geometric model of the subject’s post-surgery ipsilesional hemipelvis implant-bone assembly for use in FE simulations. This geometric model was created by combining a pre-surgery geometric model of the subject’s remaining pelvic bony anatomy with a simplified geometric model of the subject’s custom implant. Pre-surgery CT images of the subject’s pelvis were taken using a slice thickness of 0.625 mm with an in-plane pixel size of 0.78125 mm (GoldSeal Discovery CT750 HD, GE Healthcare, Chicago, IL, USA). These images were segmented using ITK-SNAP (Yushkevich and Gerig, 2017) to extract tessellated surfaces representing cortical and trabecular bone geometry. Next, the tessellated surfaces were converted into NURBS surfaces using Geomagic Wrap software (3D Systems Corporation, Rock Hill, SC, USA). The geometric model of the subject’s custom pelvic implant obtained from the manufacturer was then overlaid onto the pre-surgery cortical bone surface geometry through best-fit alignment to locate key surgical features. The surgical cutting planes in the pelvis geometric model were found by fitting planes to cut surfaces in the implant geometric model, while the locations and trajectories of the fixation screws in the pelvis geometric model were found by fitting cylinders to the screw holes in the implant geometric model. A virtual surgery was then performed in SolidWorks (Dassault Systèmes, Santa Clara, CA, USA) to dissect the pre-surgery ipsilesional pelvis geometry using these key surgical features, producing a final pelvis CAD model possessing the same bone cuts as were performed surgically.

A simplified implant CAD model that mimicked the shape of the subject’s custom pelvic implant was created to eliminate the influence of unimportant surface details on the subsequent FE analyses and increase the overall efficiency of the simulations. This simplified implant CAD model preserved engineering features of the subject’s custom pelvic implant, such as cortical flanges, screw holes, and a similarly sized and oriented acetabular cup. However, the surface of the simplified implant CAD model was created by smoothing the bone geometry mirrored from the contralesional hemipelvis.

**FIGURE 4:**
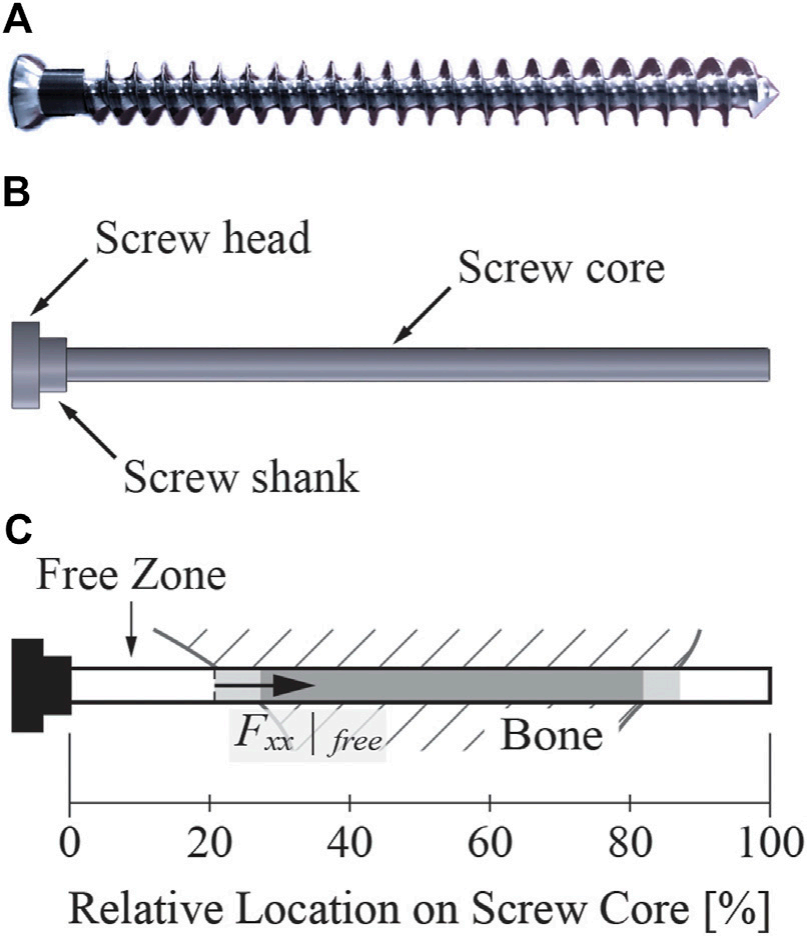
A sample screw similar to the screws used in the present study. **(A)** Image of a cancellous bone screw with the same dimensional characteristics (except for length) as the screws used in the present study. The length of the screws used in our study ranged from 15 to 80 mm. **(B)** The screw geometry used for FE modeling. The threaded part of the screw was simplified to a cylindrical screw core without the screw threads. **(C)** Illustration explaining key elements of the screw core. Regardless of the screw length, the 0% relative location on the screw core corresponded to the junction of the screw shank and core, while the 100% relative location on the screw core corresponded to the screw tip. In all figures shown in this study, the light gray shaded zone indicates the portion of the screw core where the screw was only partially embedded in bone, while the darker gray shaded zone indicates the portion on the screw core where the screw was fully embedded in the bone. The free zone indicates the portion of the screw from the beginning of the screw core to where the screw became partially embedded in bone. The axial force within the free zone was evaluated at the end of the free zone.

CAD models of the compression screws were also created. The screws were modeled as 3.0 mm wide cylinders with a 2.5-mm long and 4.9-mm wide shank and an 8.0-mm wide head, based on measurements of a similar screw made by the same screw manufacturer (Figures 4A, B). The length of each screw was obtained from surgical notes, which were referenced for making the screw geometries.

### 2.4 Pelvis-implant finite element model

An FE model of the ipsilesional hemipelvis with custom implant was built in Abaqus (Dassault Systèmes, Santa Clara, CA, USA) to simulate post-surgery implant stresses using the bone, implant, and screw CAD models described above. The FE model consisted of the implant, nine screws, the remaining iliac and ischium bone, a sphere representing the femoral head of the subject’s total hip replacement, and an intermediate liner between the acetabular cup and the femoral head (Figure 1B). The model was meshed with 10-node quadratic tetrahedral elements (C3D10; average element size: 1.73 mm), and the FE mesh contained 668,465 elements with approximately 2.9 million degrees of freedom.

Elastic material properties such as Young’s Modulus and Poisson’s Ratio were determined for all components of the FE model. Each component was assigned homogeneous material properties based on values reported in the literature with the exception of trabecular bone components (Table 1). The material properties of the trabecular bone components were heterogeneous, where Young’s modulus at each trabecular node was extracted from the subject’s pre-surgery CT imaging data. Due to the lack of a calibration phantom, we calibrated voxel-specific intensity values ( INT ) from the CT images using the air-fat-muscle calibration method (Eggermont et al., 2019; Babazadeh Naseri et al., 2021) and converted to equivalent bone mineral density ( BMD ) using $BMD\;\left[\text{gr}\cdot {\text{cm}}^{-3}\right]=0.0008\times \mathrm{INT}\;\left[\text{HU}\right]-0.8037$ (Anderson et al., 2005). The estimated BMD was then converted to bone apparent density values ( $\rho_{\text{app}}$ ) and elastic modulus ( E ) using the empirical relations shown in Equations 1, 2 (Anderson et al., 2005):

$$\rho_{app}=BMD/0.626,$$ (1)

$$E=2017.3\times {\left(\rho_{app}\right)}^{2.46}$$ (2)

**TABLE 1:** Elastic properties of FE model components.

Material	Young’s modulus (MPa)	Poisson’s ratio
Ti64 (Implant, screws, spherical femoral head) (Niinomi and Boehlert, 2015)	115,000	0.35
UHMWPE (Hip joint replacement liner) (Liu et al., 2012)	450	0.4
Cortical Bone (Böhme et al., 2012)	8,000	0.3
Trabecular Bone	mean: 661.4; range: 100–6704.7	0.3

Voxel-specific elastic modulus values were mapped to each FE node within the trabecular bone using a nearest neighbor approximation.

Physiological hip joint contact force and boundary conditions were imposed to simulate the interactions between the hemipelvis and its surrounding anatomical structures. Gait 1 and Gait 2, the two selected peaks of the post-surgery hip joint contact force, were applied as a concentrated force at the center of the femoral head. The FE model was constrained by three sets of springs as boundary conditions to represent the joints and ligaments that remained connected to the operated hemipelvis. Two sets of linear springs were defined at the sacroiliac and pubic joint surfaces to simulate the stiffness of the ligaments and cartilage at these joints. The total spring stiffness in the normal direction was 103.09 kN/mm at the sacroiliac joint and 4.24 kN/mm at the pubic joint (Li et al., 2006; Phillips et al., 2007; Hao et al., 2011; Watson et al., 2017; Dong et al., 2019). The spring stiffness in the tangential direction was set to 10% of that in the normal direction. A set of nonlinear springs was defined at the medial margin of the ischial tuberosity to represent the sacrotuberous ligament. These springs were restricted to only compress in the direction of the ligament with a total spring stiffness of 1.5 kN/mm (Phillips et al., 2007; Hao et al., 2011; Dong et al., 2019).

Two modeling approaches were used to describe the interactions between different FE model components. The first approach was a contact model that was assumed to be “hard” in the normal direction and frictionless in the tangential direction. This approach was implemented between the implant and cut bone surfaces, which were free to come into contact with or separate from each other. The second approach was a tied constraint where the constrained surfaces were bonded together without separation. This approach was implemented between the femoral head and liner of the total hip replacement and between the liner and the custom implant acetabular cup.

Several important concepts must be explained briefly to facilitate the subsequent description of the six different screw modeling methods. “Free zone” of a screw refers to the section of the screw core that does not contact bone, located right before the screw begins to enter the bone (Figure 4C). The axial force within the free zone of each screw was evaluated at the end of the free zone, away from the screw shank. “Pretension step” refers to the first step of a two-step FE simulation. The goal of the pretension step is to preload the screws such that the axial forces within the screw free zones closely match their corresponding desired pretension forces at the end of the step. The process of inferring the magnitude of the desired pretension forces for the screws is described in detail following the description of the various screw modeling methods. During the pretension step, no external forces were applied so that the step resembled tightening of the screws. “Simulation step” refers to the second step of the two-step FE simulation. During the simulation step, external loads are applied to the FE model to simulate the stress distribution within the screw or its surrounding structures. Specifically, hip joint contact forces were applied during the simulation step.

**FIGURE 5:**
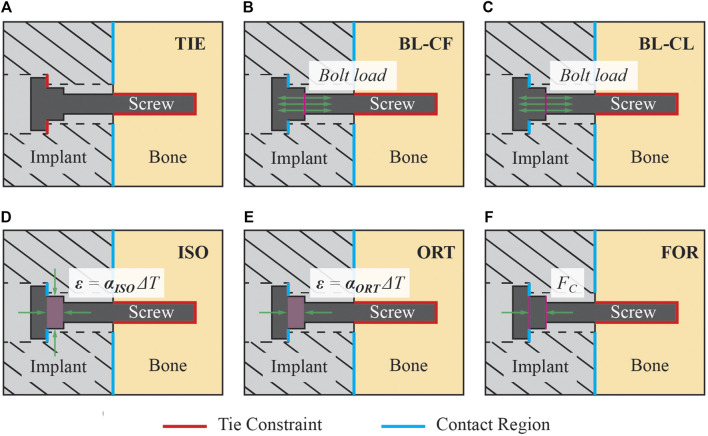
Description of the six screw modeling methods used in the present study. **(A)** Modeling method TIE used tie constraints for all interactions between the screw and its surrounding structures. This modeling method was the only one that used a tie constraint between the screw head and the implant, while the other five modeling methods used a contact model instead. **(B)** Model BL-CF applied the pretension force as a bolt load to the screw core during the pretension step. During the simulation step, this modeling method propagated the bolt load from the pretension step and kept its magnitude constant. **(C)** Model BL-CL applied the pretension force as a bolt load to the screw core during the pretension step. During the simulation step, this modeling method fixed the bolt axial length at its current length inherited from the pretension step. **(D)** Modeling method ISO induced the pretension force by introducing a pseudo-thermal field within the screw shank, which was subjected to an isotropic thermal contraction. **(E)** Modeling method ORT also induced the pretension force using a pseudo-thermal field within the screw shank but subjected it to an orthotropic thermal contraction in only the axial direction of the screw. **(F)** Modeling method FOR induced the pretension force by applying a set of equal and opposite compressive forces to the nodes on the screw shank circular surfaces.

Six distinct FE screw modeling methods were implemented and evaluated for fixation durability analysis (Figure 5). The six screw modeling methods differed in various aspects. The first screw modeling method, named “TIE”, differed from the remaining five screw modeling methods in two major aspects (Table 2). First, the TIE modeling method used Abaqus built-in functionality to define tied constraints between the screws and all neighboring components (*i.e.,* cut bone surfaces and the implant). In contrast, the five remaining screw modeling methods used contact models between screw heads and corresponding screw holes in the implant and tied constraints only between the screws and cut bone surfaces. Second, the TIE modeling method did not preload the screws to estimate the stress distributions within the fixation screws and hence did not require a pretension step before the simulation step. In contrast, the remaining five screw modeling methods required the pretension step to preload the screws as a part of the two-step FE analysis process.

**TABLE 2:** Differences in solution process between the screw modeling methods.

Model	Abaqus built-in	Pretension step	Iterative process
TIE	Yes	No	N/A
BL-CF	Yes	Yes	No
BL-CL	Yes	Yes	No
ISO	No	Yes	Yes
ORT	No	Yes	Yes
FOR	No	Yes	Yes

Two of the five remaining screw modeling methods, named “BL-CF” and “BL-CL,” used Abaqus built-in Bolt Load functionality to apply axial forces directly to the screw core. These two methods differed in how the loading of the bolt load was defined during the simulation step. The BL-CF methods propagated the bolt load from the pretension step and kept the bolt load magnitude constant during the simulation step, while BL-CL method fixed the axial length of the bolt at its current length inherited from the pretension step and allowed the axial force within each screw to change according to the response of the model.

The three remaining screw modeling methods, named “ISO”, “ORT”, and “FOR”, utilized known physical relations to induce the pretension force. Among them, the ISO and ORT modeling methods induced the pretension force within the screw free zone by introducing thermal strain resulting from a pseudo temperature decease caused by a pseudo temperature field defined within the screw shank. The pseudo temperature decrease induced a thermal contraction of the screw shank, which subsequently imposed a compressive force in the screw core. Because the extent of pseudo temperature decrease dictated the magnitude of the induced axial force, the respective pseudo temperature changes of all screws were calibrated through an iterative procedure to generate the desired axial forces. While these two screw modeling methods were similar, they differed in the degrees of freedom for the thermal contraction effect, which as isotropic for the ISO modeling method but limited to only the screw axial direction for the ORT modeling method. Different from the ISO and ORT modeling methods, the FOR modeling method induced the pretension force within the screw free zone by introducing mechanical strain resulting from a set of two equal and opposite compressive forces applied to the screw shank. The compressive forces were applied to two reference nodes, defined at the center of each of the two circular surfaces of the screw shank and attached to the nodes on their respective surfaces through rigid body connections. Like the ISO and ORT modeling methods, the FOR modeling method required an iterative calibration procedure to find the necessary magnitude of the compressive force to generate the desired pretension force. These modeling methods are henceforth called the “iterative” methods.

The magnitude of the pretension force $F_{P}$ to be induced within the screw free zone was inferred through a series of steps that involved estimating the optimal insertion torque $T_{ins}$ , the associated stripping torque $T_{str}$ , and the pullout force failure threshold $F_{PO}$ for each screw. First, the pullout failure threshold $F_{PO}$ for each screw was estimated using the published empirical relationship shown in Equation 3 (Chapman et al., 1996):

$$F_{po}=S\times L\times \pi \times D_{major}\times TSF,$$ (3)

where S is the bone’s ultimate shear stress, L is the length of thread engagement in the material, $D_{major}$ is the major diameter of the screw, and TSF is the dimensionless thread shape factor. The ultimate shear stress for trabecular bone was calculated using an empirical correlation between the compressive ultimate strength (Ebbesen et al., 1997; Fleps et al., 2020) and the apparent density of the ilium and a 0.44 shear-to-compressive strength ratio (Sanyal et al., 2012). Hence, the relationship between the ultimate shear stress of the trabecular bone and the corresponding bone apparent density was defined as $S=0.44\times \sigma_{u,c}=0.44\times 19.08\times {\sigma_{\text{app}}}^{2.15}$ , where $\sigma_{\text{app}}$ was the average apparent density extracted from the subject’s CT images, which was 0.422 g/cm^3^. The TSF of a screw was defined as $TSF=\left(0.5+0.57735d/p\right)$ , where d is the thread depth defined as $\left(D_{major}-D_{\left.minor\right)}/2\right.$ , $D_{minor}$ is the minor diameter of the screw, and p is the thread pitch.

For each screw, the calculated value of $F_{PO}$ was used to estimate an associated optimal insertion torque $T_{ins}$ for each screw. Although the actual insertion torque for each screw was not measured at the time of the surgery, the optimal insertion torque was approximated as being 65% of the predicted stripping torque ( $T_{str}$ ) of each screw (Lawson and Brems, 2001). The optimal insertion torque of each screw was then estimated through Equations 4, 5 by first calculating the stripping torque $T_{str}$ (Fletcher et al., 2019):

$$T_{\text{str}}=F_{\text{PO}}\times r\times \frac{p+2\mu r}{2r‐\mu p},$$ (4)

$$T_{ins}=0.65\times T_{str},$$ (5)

where r is the pitch radius of the screw and μ is the coefficient of friction of the bone-screw interface. The pitch radius r was defined as $\left(D_{major}+D_{\left.minor\right)}/4\right.$ .

Finally, the optimal insertion torque $T_{ins}$ for each screw was converted to an equivalent inferred pretension force $F_{P}$ to be induced within the free zone of the screw during the pretension step of each FE simulation. The relationship between the insertion torque $T_{ins}$ and inferred pretension force $F_{P}$ in a screw was described by Equation 6 (Deutsches Institut für Normung, 1991; Shoberg, 2000):

$$F_{P}={\;T}_{ins}\;/\left(0.159\times p+0.578\times 2r\times \mu_{G}+\frac{d_{Km}}{2}\mu_{K}\right),$$ (6)

where $\mu_{G}$ is the coefficient of friction in the threads, $\mu_{K}$ is the coefficient of friction under the head, and $d_{Km}$ is the mean bearing diameter under the head. The coefficient of friction $\mu_{G}$ between machined Ti64 alloy and trabecular was set to 0.42 (de Vries et al., 2022), while the coefficient of friction between $\mu_{K}$ two titanium surfaces was set to 0.441 (Guda et al., 2008). The pretension force was calculated for each of the nine fixation screws. Theoretically, at the end of the pretension step, each screw experienced an axial force whose magnitude was the screw’s corresponding inferred pretension force.

Because the pretension force of each screw could not be implemented directly in the iterative modeling methods (*i.e.*, ISO, ORT, and FOR), a root-finding procedure based on the Secant Method was employed to determine the necessary temperature change or compressive force required in the screw shank to induce the inferred pretension force in the screw free zone. In the Secant Method, two sets of initial guesses are required to begin the iterative procedure. The equation for the next iteration is $x_{n+1}=x_{n}-f\left(x_{n}\right)∙\frac{x_{n}-x_{n-1}}{f\left(x_{n}\right)-f\left(x_{n-1}\right)}$ , where $x_{n}$ is the magnitude of current temperature change or compressive force, and $f\left(x_{n}\right)$ is the difference between the induced axial force in a screw in the current step ( $F_{n}$ ) and the inferred pretension force ( $F_{P}$ ). A single-screw FE model was built to generate the initial guesses for the necessary temperature changes or compressive forces (see Supplementary Material for details). The temperature changes or compressive forces that caused each single-screw FE model to produce the specified pretension force were used as the initial guesses ( $x_{0}$ ) for the hemipelvis FE model with all nine screws included. The Secant Method pretension step was then run using the hemipelvis FE model with the guessed temperature changes or compressive forces determined for each screw separately and iterated until the induced axial forces in all nine screws were within 5% of the desired pretension forces.

After the iterative process was completed, the simulation step of the FE model was run to simulate the stress distributions in the screws and compute the simulated axial force within the free zone. During the simulation step, the conditions used to induce the pretension forces were maintained, and the hip joint contact force was applied. All hemipelvis FE analyses were simulated using an 8-core, 16-processor, 3.70-GHz PC workstation. Additionally, a mesh sensitivity study was conducted to gain insight into the influence of mesh refinement on stress concentrations for the three iterative screw modeling methods (see Supplementary Material for details).

### 2.5 Screw failure evaluation

All nine screws in the hemipelvis FE model were evaluated for the likelihood of two failure modes–pullout and high-cycle fatigue. The simulated axial force and peak von Mises stress for each of the nine screws was computed for all combinations of six screw modeling methods and two load cases. The pullout failure threshold computed using Equation 3 was compared to the simulated axial force experienced by each screw to determine the likelihood of screw pullout (Figure 4C). The fatigue limit of the Ti64 screw was estimated and compared to the simulated peak von Mises stress experienced by the core of each screw to determine the likelihood of high-cycle fatigue failure. The von Mises stress distributions within the first 3 mm (equivalent to the core diameter) of the screw core were neglected to avoid stress concentrations. The fatigue limit of the screws was estimated by first determining the fatigue limit of a standard Ti64 alloy rotary beam test specimen from the relationship between cyclic stress magnitude and number of cycles to fatigue failure (Niinomi, 2008). The fatigue limit of the screws was then computed from the fatigue limit of the standard specimen using the Marin equation as shown below in Equation 7 (Marin, 1962; Budynas and Nisbett, 2011):

$$S_{e}=K{S_{e}}^{\prime },$$ (7)

where $S_{e}$ and ${S_{e}}^{\prime }$ are the fatigue limits of the screw and rotary-beam test specimen, respectively, and K is the product of modifying factors (e.g., surface factor, gradient factor, load factor, temperature factor).

## 3 Results

### 3.1 Inferred pretension forces and failure criteria

The estimated pullout force failure thresholds for the nine screws produced a wide range of inferred screw pretension forces (Table 3). Pullout force failure thresholds ranged from 221.4 N for Cortical 1, 2, and 3 screws to 1060.9 N for Cancellous 2 screw. The associated pretension forces ranged from 149.7 N for Cortical 1, 2, and 3 screws to 717.5 N for Cancellous 2 screw. The ratios between the pullout force failure threshold to inferred pretension force was 1.48 for all screws by design. The fatigue limit of the Ti64 screws was calculated to be 542.2 MPa.

**TABLE 3:** Inferred pullout force failure threshold $F_{PO}$ , optimal insertion torque $T_{ins}$ , and pretension force $F_{P}$ for each screw.

Screw	$F_{PO}$ [N]	$T_{ins}$ [N-m]	$F_{P}$ [N]
Cancellous 1	1026.7	2.1	694.4
Cancellous 2	1060.9	2.2	717.5
Cancellous 3	295.2	0.6	199.6
Cortical 1	221.4	0.5	149.7
Cortical 2	221.4	0.5	149.7
Cortical 3	221.4	0.5	149.7
Cortical 4	312.3	0.6	211.2
Cortical 5	393.6	0.8	266.2
Cortical 6	702.6	1.4	475.2

### 3.2 Screw failure analyses

**FIGURE 6:**
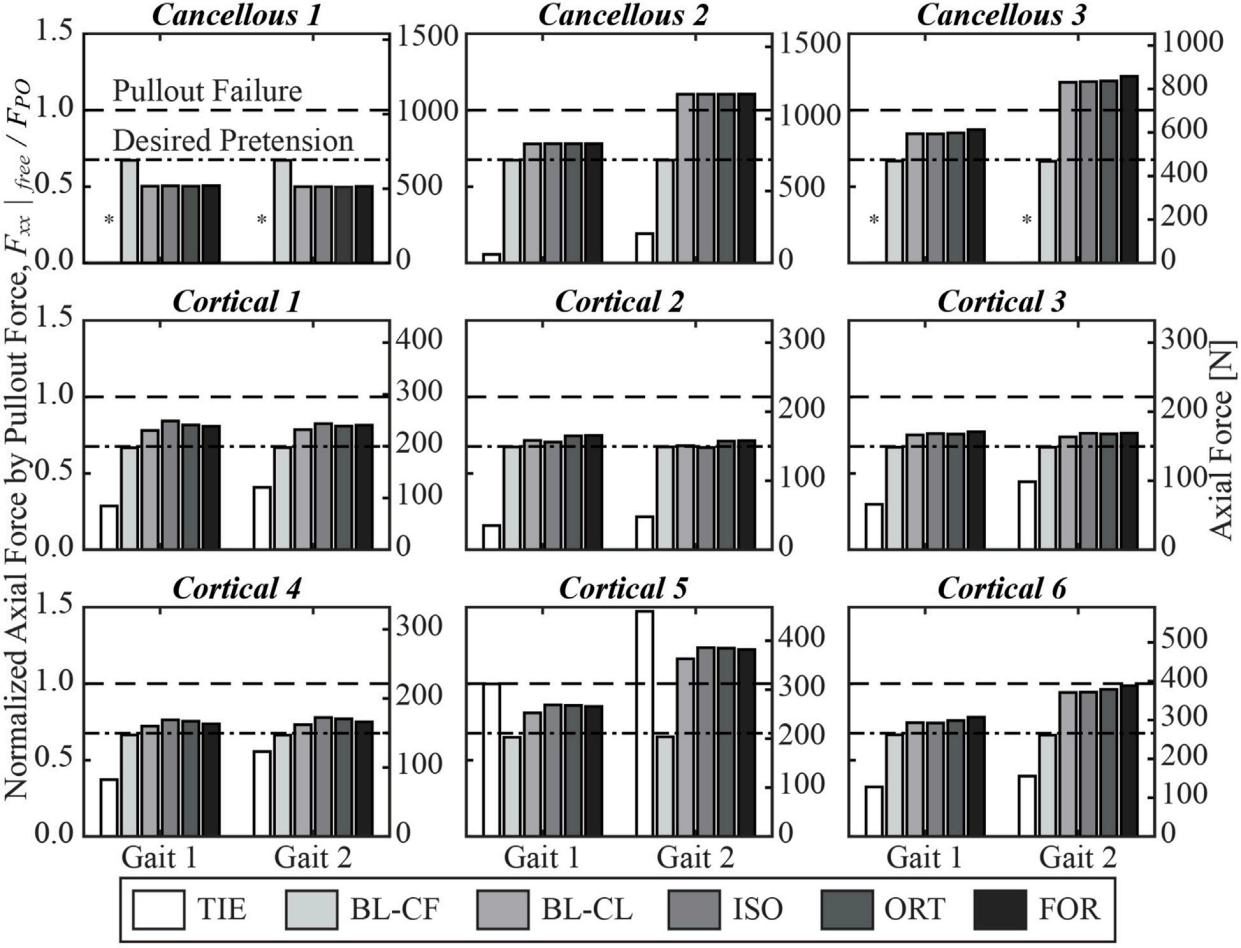
Axial force in the free zone for each screw at the two selected locations in the gait cycle, normalized by the pullout failure threshold. A “*” denotes that the simulated axial force in the free zone was below zero and was therefore compressive instead of tensile.

The three iterative screw modeling methods and the BL-CL method all produced similar axial force predictions in the screw free zones (Figure 6). Screw axial forces predicted by the BL-CF modeling method were close to those predicted by these four methods, while predictions from the TIE method being the most dissimilar. For the three iterative screw modeling methods and the BL-CL method, some screws (i.e., Cancellous 1 and Cortical 1 through 4) experienced similar predicted axial forces at the two locations in the gait cycle, with the predictions being well below the pullout failure limit. In contrast, other screws (i.e., Cancellous 2 and 3 and Cortical 5 and 6) experienced different axial forces at the two locations in the gait cycle, with the larger forces at the second location being near or above the pullout failure limit.

**FIGURE 7:**
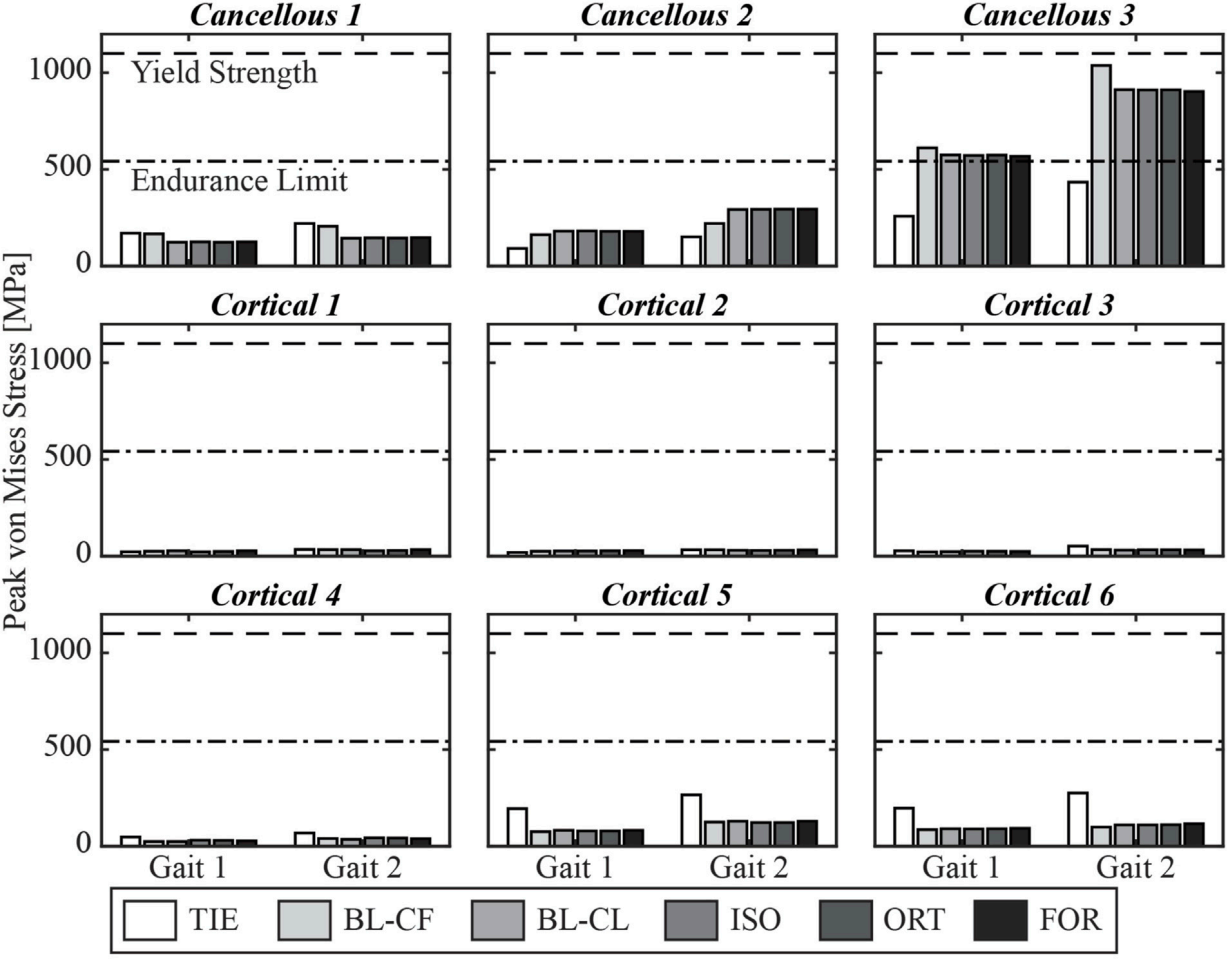
Simulated peak von Mises stress for each screw at the two selected locations in the gait cycle.

Furthermore, the three iterative screw modeling methods and the BL-CL method all produced similar peak von Mises stress predictions in the screw cores (Figure 7). Peak von Mises stresses predicted by the BL-CF modeling method were similar to those predicted by these four methods, while predictions from the TIE method remained dissimilar. All screws except for Cancellous 3 were predicted to experience peak von Mises stress below the screw fatigue limit during level-ground walking, regardless of the selection of the screw model. This finding suggested that all screws except for Cancellous 3 could sustain long-term cyclic loading without failing. In contrast, different screw modeling methods produced different predictions for the likelihood of Cancellous 3 fatigue failure. For all screw modeling methods apart from the TIE method, the Cancellous 3 screw had a peak von Mises stress that was above the fatigue limit, making the screw susceptible to fatigue failure. However, for the TIE modeling method, the Cancellous 3 screw had a peak von Mises stress that was below the fatigue limit, suggesting that it was not susceptible to fatigue failure.

### 3.3 Inferred pretension forces

**FIGURE 8:**
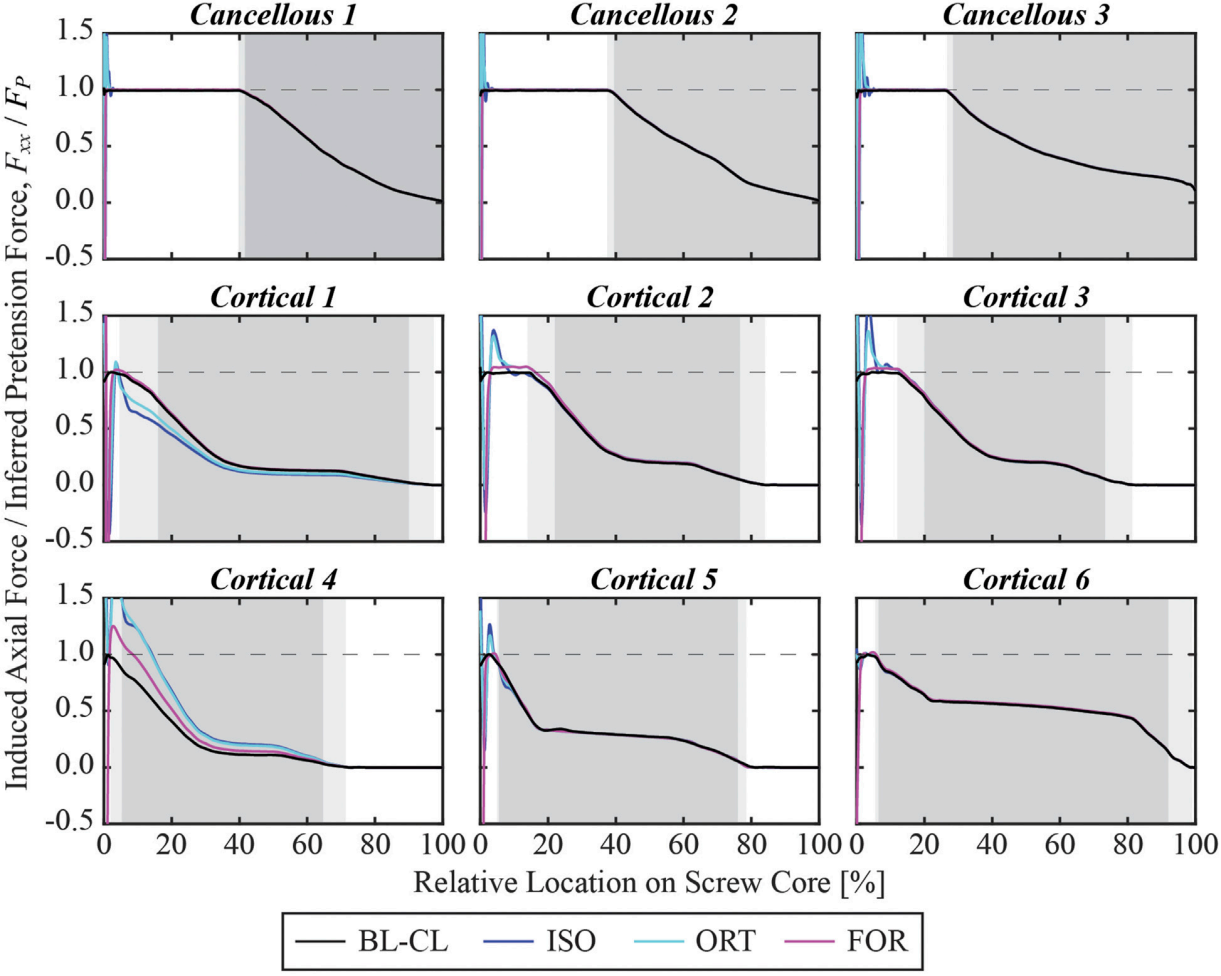
Simulated axial force profile for each screw within its core normalized by the inferred pretension force at the end of the pretension step. The 0% relative location denotes the beginning of the screw core, which was adjacent to the screw shank, while the 100% relative location denotes the screw tip. The light gray shaded zone denotes the region where the screw is partially embedded in the bone, and the dark gray shaded zone denotes the region where the screw is fully embedded in the bone.

**FIGURE 9:**
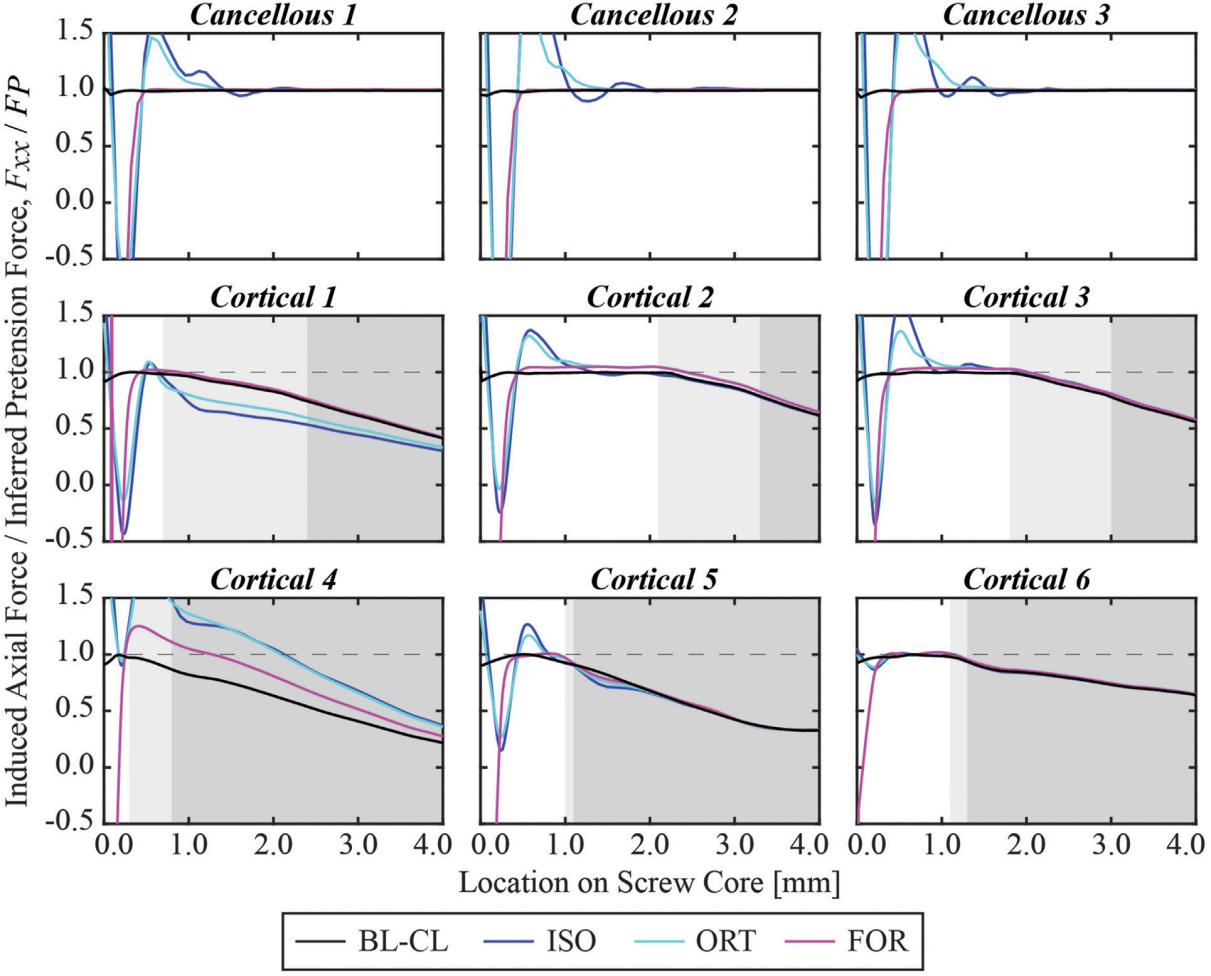
Simulated axial force profile for each screw within the first 4 mm of its core normalized by the pretension force at the end of the pretension step.

At the end of the pretension step, the induced screw axial force within the free zone matched the inferred pretension force. The axial force profiles generally exhibited oscillations at the beginning of the screw core, which stabilized at a distance and decreased once the screw began entering the bone (Figure 8). For the cancellous screws, the axial forces converged to the inferred screw pretension forces within the first 3 mm and maintained their magnitude for the rest of the screw free zone (Figure 9). However, due to the shorter length of the free zone within cortical screws, the axial force in these screws either did not have a plateau region where the magnitude was maintained or had a much shorter plateau region before the screw entered the bone. For all nine screws, the axial force profiles simulated using the BL-CL modeling method exhibited the fewest oscillations. The BL-CL modeling method also required the shortest distance for the axial force to plateau without exhibiting stress concentrations, followed by the FOR modeling method. The mesh sensitivity study revealed that at least one layer of elements was required to eliminate stress concentration effects when using the FOR method, two layers of elements when using the ORT method, and 3 mm when using the ISO method (see Supplementary Material for details).

### 3.4 Computation time

**FIGURE 10:**
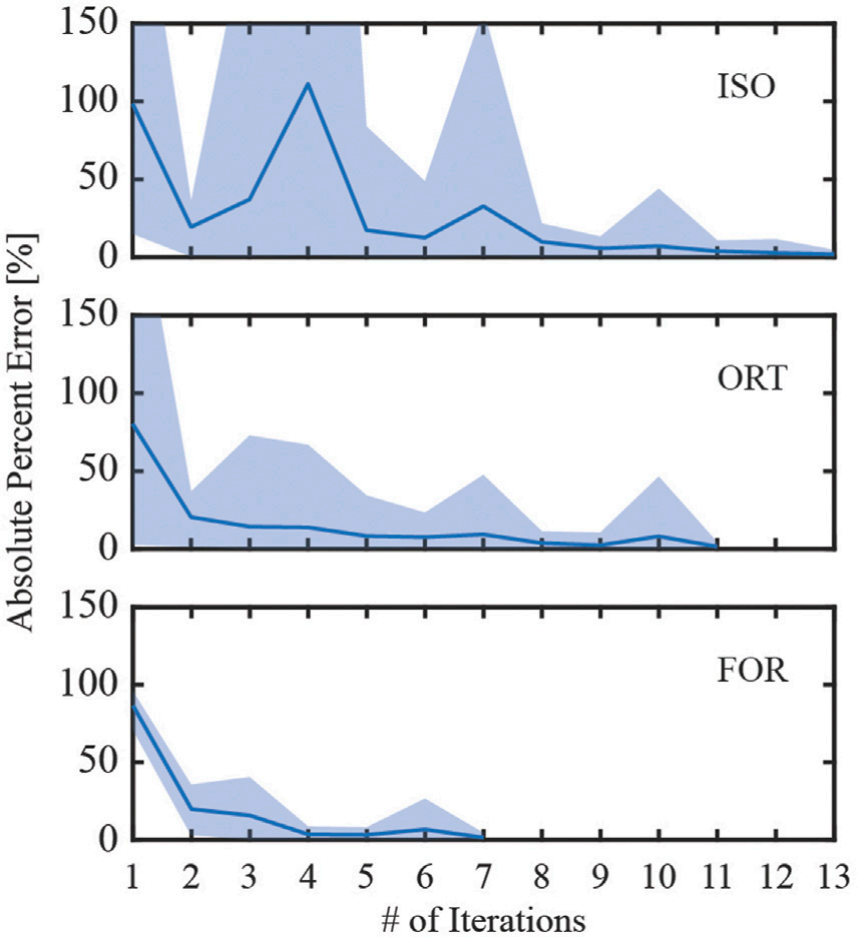
Absolute percent error during the iterative process used to induce the inferred pretension force in the free zone of all screws. The solid line denotes the mean absolute percent error of the induced pretension force compared to the inferred pretension force across all nine screws, while the shaded region denotes the range of absolute percent errors. The iterative process ended when the maximum percent error for the iteration was below 5%.

Among the three iterative screw modeling methods, the FOR method required the fewest iterations to reach a 5% error in inferred pretension forces for all nine screws (Figure 10). These three modeling methods began their first iteration with a comparable yet sizable mean absolute percent error. The mean absolute percent errors for the inferred pretension force compared to the pretension force at the end of the iterative process were between 1.4% and 1.9%. The time spent running the iterative process for the three modeling methods ranged from 35.5 to 64.4 h before the simulation step began (Table 4). The FOR model was the most time efficient among the three iterative modeling methods, followed by the ORT method and the ISO method. For comparison, the BL-CL method did not require an iterative process to complete the pretension step. Consequently, the BL-CL modeling method required an order of magnitude less total computation time (pretension + simulation steps) than did the three iterative modeling methods.

**TABLE 4:** Computation time necessary for each screw model using an 8-core, 16-processor, 3.70-GHz PC workstation.

Model	Pretension step		Simulation step		Full analysis
	# Of iter	Accumulative step Run time [h]	Step run time [h]		Total run time [h]
			*Gait 1*	*Gait 2*	*Pretension + Gait 1 + Gait 2*
TIE	N/A	N/A	3.7	3.8	7.5
BL-CF	N/A	5.1	2.2	6.5	13.8
BL-CL	N/A	5.0	1.9	3.0	9.9
ISO	13	64.4	2.1	5.4	71.9
ORT	11	57.6	2.1	3.2	62.9
FOR	7	35.5	2.0	3.3	40.8

## 4 Discussion

This study evaluated six different FE screw modeling methods for predicting compression screw pullout and fatigue failure in a custom pelvic implant. Three modeling methods (tied constraints (TIE), bolt load with constant force (BL-CF), and bolt load with constant length (BL-CL)) generated screw axial forces using functionality built into Abaqus FE software, while the remaining three modeling methods (isotropic pseudo-thermal field (ISO), orthotropic pseudo-thermal field (ORT), and equal-and-opposite force field (FOR)) generated screw axial forces using iterative physics-based relationships that can be implemented in any FE software. The ability of all six modeling methods to match specified screw pretension forces and predict screw pullout and fatigue failure was evaluated using an FE model of a custom pelvic implant with a total hip replacement, where applied hip contact forces were estimated at their two peaks in the gait cycle. For each of the nine screws in the custom implant FE model, the likelihood of screw pullout and failure were predicted using maximum screw axial force and maximum von Mises stress, respectively. The three physics-based iterative modeling methods and the Abaqus BL-CL method produced nearly identical predictions for likelihood of screw pullout and fatigue failure, giving us confidence in the results produced by these methods. In contrast, the other two built-in Abaqus modeling methods yielded vastly different predictions. The Abaqus BL-CL method required the least computation time, largely because no iterative process was needed to induce specified screw pre-tension forces. Of the three iterative methods, FOR required the least number of iterations and thus the least computation time. These findings suggest that the choice of screw modeling method can drastically alter FE-based conclusions regarding the likelihood of pullout and fatigue failure for compression screws used in custom pelvic implants.

In contrast to previous FE studies of custom pelvic implants, the current study used heterogenous pretension forces across the screws. Previous studies used the BL-CL modeling method, or some variation thereof, to analyze the stress distributions in or around compression screws used with custom pelvic implants (Dong et al., 2018; Maslov et al., 2021; Soloviev et al., 2023). Each of these studies attempted to identify optimal screw pretension forces for their respective bone-implant assemblies. In each study, a single arbitrarily selected axial force ranging from 0 to 3000 N was applied to all screws to determine which level of pretension force was the most appropriate to avoid mechanical failures of the implant, screws, or remaining pelvic bone. Dong et al. (2018) found that the screw pretension force should be kept below 1000 N to avoid screw fatigue failures, while Maslov et al. (2021) and Soloviev et al. (2012) reported that the optimal pretension force should be between 500 and 1000 N, and preferably around 500, to avoid bone fracture. However, the screws used in these studies differed in size and location from the ones used in the present study. Given the wide size (15–80 mm in core length) and location differences for the screws used in our study, we felt that it would be unrealistic to apply the same pretension force to all screws. In the absence of screw pretension measurements, we inferred screw pretension forces using widely accepted empirical relationships reported in the literature. Various screw geometric parameters, model component material properties, and friction coefficients between components were required to estimate the pretension force for each screw. The force to be induced during the pretension step was ultimately calculated using a specified fraction of the stripping torque (*i.e.*, the assumed optimal insertion torque). The inferred pretension forces for the screws in our study varied from 149.7 to 717.5 N, all within the range of suggested pretension forces provided by previous studies (Dong et al., 2018; Maslov et al., 2021; Soloviev et al., 2023). The same conversion process from the assumed optimal insertion torque to pretension force was used in previous studies, particularly of dental implants, which typically consisted of a single compression screw (Lang et al., 2003; Dannaway et al., 2015; Satpathy et al., 2022). However, this “optimal” insertion torque was only validated to be optimal for a single screw to resist axial forces. In a complex bone-implant system with multiple screws, such as modeled in the present study, the magnitude of optimal insertion torque is not well understood. Even if optimal insertion torques could be calculated, in reality it is difficult for surgeons to “feel” the precise amount of torque being applied without the use of a torque wrench. In the future, surgeons may want to consider using a torque wrench in the operating room to apply the desired amount of torque to each screw. However, even with a torque wrench, it would be difficult to achieve the desired screw forces in a multi-screw implant system due to the “elastic interaction effect.” This effect causes the tightening of one screw in a multi-screw system to affect the pretension forces in all other screws that were previously tightened. During surgery, since the screws would be tightened in a sequence, the screws that were tightened to their optimal insertion torque might be affected by the tightening of neighboring screws. Investigating the effect of screw-tightening sequence was beyond the scope of the present study. The FE model in our study was preloaded with the carefully approximated pretension forces and represented an idealized scenario where all screws were subjected to an assumed optimal pretension force.

While the BL-CL modeling method has been used in previous studies, limited information is available to explain how Abaqus implements bolt loads as pretension forces. The reliability of the defined bolt loads had not been verified in any FE studies of custom pelvic implants. Due to the built-in status of the Abaqus BL-CL functionality, it cannot be used in other FE software packages. In this study, the maximum axial force and peak von Mises stress for each screw predicted by the BL-CL modeling method were highly consistent with predictions generated using the ISO, ORT, and FOR modeling methods, which are based on well-understood physics relationships. Thus, for FE software other than Abaqus, ISO, ORT, and FOR modeling method are viable alternatives.

Though the TIE modeling method has also been used in similar studies, it did not predict realistic interactions between the compression screws and custom pelvic implant used in this study. During surgery, the surgeon tightens the compression screws so that the screw heads compress the implant against remaining bone. However, the use of tie constraints prevented the screw heads from separating from the implant, and thus the stress on the screws was partially transferred to the implant or *vice versa*. In reality, it is possible for the head of a compression screw to become separated from the implant. Because of this deviation from physical reality, the peak von Mises stresses and axial forces predicted by the TIE modeling method were substantially different from those predicted by a modeling method involving a contact model between the screw heads and the custom implant. Though not appropriate for the present study, the TIE model could nonetheless be suitable for modeling “locking” screws that are threaded into the holes of the custom pelvic implant. In a previous study (Zhu et al., 2023), the TIE modeling method was used to analyze the fixation durability of a custom implant, and the simulations successfully identified the compromised screws and their failure modes. The screws in that study were all locking screws which were modeled as inseparable from the implant through the use of tie constraints. Had the screws used in the present study been locking screws, the simulations with the TIE model would likely have been sufficient to assess screw fixation durability.

Although the BL-CF and BL-CL modeling methods differed only in the bolt loading methods used during the simulation step, the BL-CF method produced markedly different and less realistic simulation results. In the BL-CF method, the magnitudes of the imposed bolt loads on the screws during the simulation step were propagated from the pretension step and kept constant thereafter. Indeed, the axial forces in all screws predicted with the BL-CF method were within 2% of the inferred pretension forces. This observation suggested that the applied bolt loads in the BL-CF method acted as constraints on the screws in the axial direction so that the axial forces were maintained at a similar magnitude to the applied bolt load magnitude. Non-physical boundary conditions might have been imposed on the FE model to maintain the constant axial forces during this step. Hence, the BL-CF method always failed to provide realistic axial forces on the screws. Furthermore, the reliability of the simulated von Mises stress was questionable as well. Although the von Mises stresses predicted with the BL-CF method were generally comparable to those predicted with the iterative physics-based modeling methods and the BL-CL method, the peak von Mises stress of Cancellous 3 predicted with BL-CF was close to the material yield strength and more than 13% higher than that predicted with the iterative physics-based modeling methods. This notable difference in von Mises stress predictions was likely the result of excessive axial stress introduced by the non-physical boundary conditions imposed to maintain each axial force at a constant value. The inferior performance of the BL-CF method highlights the importance of selecting the most appropriate modeling method for the physical situation.

Although no *in vivo* measurements of von Mises stress or axial force were available for comparison with our simulation results, the clinical records for the subject being modeled provided important insights into the accuracy of the failure predictions produced by the different screw modeling methods. Specifically, the Cancellous 3 screw experienced a pullout failure. Consequently, the subject went through revision surgery to reinforce the fixation of this screw. Neither the TIE nor the BL-CF modeling method was able to predict the pullout failure of Cancellous 3, while the BL-CL, ISO, ORT, and FOR modeling methods all predicted that Cancellous 3 was susceptible to pullout failure. However, the simulations performed using the latter methods also predicted that Cancellous 3 was susceptible to fatigue failure and Cortical 5 to pullout failure, which did not occur. For Cancellous 3, it is possible that the pullout failure occurred before the potential fatigue failure could occur. For Cortical 5, even if the screw pulled out, it would be difficult to visualize the failure. Unlike for Cancellous 3, which was the only screw used in the pubic region, Cortical 5 was one of eight screws used in the ilium region. Thus, the remaining screws would maintain fixation visually even in the absence of Cortical 5. It is also worth noting that the simulations predicted potential failures at a single snapshot in time and provided no information about a possible sequence of failures.

**FIGURE 11:**
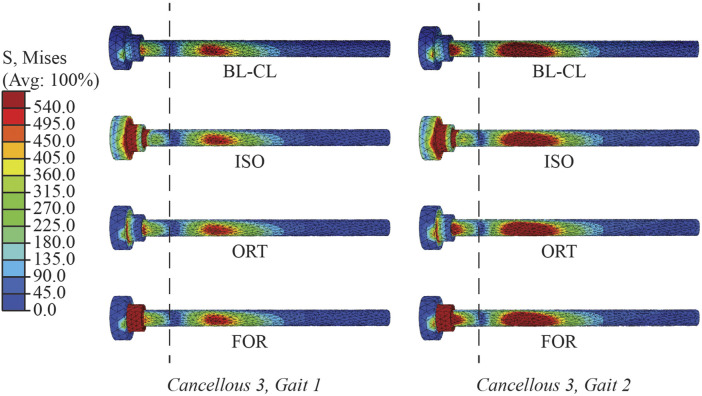
Von Mises stress distributions in the Cancellous 3 screw simulated using BL-CL, ISO, ORT, and FOR modeling methods at the end of the simulation step for both load cases. The dashed lines divide the screw at 3 mm away from the shank. The stress distributions to the right of the dashed lines were similar in magnitude and pattern for all four of these modeling methods with both load cases.

Many similarities existed among the BL-CL, ISO, ORT, and FOR modeling methods. The four methods predicted similar von Mises stress and axial force profiles within the screw core except for the first 3 mm near the screw head. Regardless of the chosen screw modeling method, when the influence of the stress concentration was excluded, the von Mises stress was undisturbed beyond 3 mm away from the screw shank. In this region, the von Mises stress distributions predicted by the four screw modeling methods appeared similar, and the peak von Mises stresses for each screw were within 6.2% on average from each other (Figure 11). In contrast, outside this region near the screw head, the von Mises stress distributions were dissimilar due to different types of strains being used to induce pretension force and relatively erratic due to the presence of stress concentrations near sharp corners. As such, the ability to resolve stress concentrations was particular important and was closely tied to the reliability of a screw modeling method.

Compared to the three iterative screw modeling methods, the BL-CL method was superior in two ways. First, because an iterative process was not necessary for the BL-CL method, it took the least amount of computation time to induce the inferred pretension forces during the pretension step. Second, given the same element size, the BL-CL method required the shortest distance at the beginning of the screw core to resolve stress concentrations. These advantages allowed the BL-CL method to provide reliable stress predictions over a longer span of the screw core. If the Abaqus built-in bolt load functionality is not available, our results suggest that the FOR method is the best alternative. Among the iterative modeling methods, the FOR method required the least amount of simulation time and required the shortest distance at the beginning of the screw core to resolve stress concentrations. The mesh sensitivity study further confirmed the FOR method’s superior ability to resolve stress concentrations.

When using one of the iterative modeling methods, the length of the free zone relative to the length of the stress concentration zone in each screw could have a substantial influence on the accuracy of the predicted axial forces. Because the axial forces were evaluated at the end of the screw free zone, for certain cortical screws, the axial forces were measured within the 3 mm zone where stress concentrations were present. The influence of stress concentrations was particularly evident in the induced pretension force profile for Cortical 4. The length of the screw free zone was less than half a millimeter. At an average element size of 0.8 mm within the screws, this screw free zone length was shorter than the minimum distance (*i.e.*, one element size) necessary to move beyond the stress concentrations regardless of the iterative screw modeling method being used. Consequently, instead of having a plateaued profile before exiting the screw free zone and a decreasing profile thereafter, the induced pretension force in Cortical 4 peaked within the partially embedded region of the screw at around 0.5 mm away from the beginning of the screw core. Unlike the induced pretension force profiles in most of the other screws, which were generally similar in shape and magnitude across the three iterative screw modeling methods, the induced pretension force profile in Cortical 4 predicted with the ISO and ORT methods was notably elevated compared to that predicted with the FOR method. Similarly, Cortical 1 also had a relatively short free zone (about 0.7 mm long) compared to the other screws. Consequently, Cortical 1 had relatively dissimilar axial force profiles across the different modeling methods compared to other screws. The short free zone made calibration of the induced pretension force more likely to be influenced by stress concentrations, causing the rest of the screw to be inaccurately loaded during the simulation step. For this reason, the predicted axial forces for the screws with a shorter free zone, such as Cortical 1 and Cortical 4, were not as reliable as for screws with a longer free zone, such as the cancellous screws. In the future, cortical screws could be meshed with finer elements to alleviate stress concentrations within a shorter distance, thereby producing more accurate axial forces.

The necessary computational runtime for the three iterative screw modeling methods presented another challenge for predicting fixation durability. At a minimum, the iterative process required 35.5 h to complete using an 8-core, 16-processor, 3.70-GHz PC workstation. In cases where use of the BL-CL model is not feasible, the runtime necessary to complete the iterative process could be prohibitive given the short timeframe available clinically for designing custom pelvic implants for sarcoma patients. However, in the authors’ opinion, the additional simulation time would be worthwhile if the long-term durability of a patient’s custom pelvic implant could be improved.

Given the modeling and computational complexity of the present study, a number of important limitations should be kept in mind. First, because of limitations in computational speed, we chose to model bone as a linear elastic material, even though bone has been shown to behave as a nonlinear viscoelastic material (Morgan et al., 2018). In particular, when subjected to successive stress relaxation cycles during experimental bone screw pullout testing, the nonlinear viscoelastic behavior of bone can reduce bone screw pullout strength by approximately 20% (Inceoglu et al., 2006). This observation indicates that the inferred pullout force calculated for each screw (Table 3) is likely an overestimate. Consequently, two screws (Cancellous 3 and Cortical 5) that had a high likelihood of pullout failure for the Gait 2 condition would have a high likelihood for the Gait 1 condition as well (Figure 6). In addition, one screw (Cortical 6) that was just below the threshold for pullout failure would move above the threshold for the Gait 2 condition (Figure 6). Thus, at least for the present study, we do not believe that simulating repeated stress relaxation loading cycles with bone modeled as a nonlinear viscoelastic material would have changed our predictions of likelihood of screw pullout failure substantially.

Second, the use of computationally estimated rather than experimentally measured hip joint contact forces in the FE model may have affected our results. The neuromusculoskeletal model used in our study was modified from an OpenSim model that had previously been calibrated using the same subject’s pre-surgery motion, ground reaction force, and EMG data. Although several changes were made to this model to reflect the subject’s post-surgery anatomy, muscle attachment sites and muscle-tendon model parameter values for the retained muscles were assumed to remain unchanged from the pre-surgery model. This assumption was the most questionable for muscles that were detached and later reattached during surgery. Though pre-surgery muscle-tendon properties may not accurately describe the post-surgery condition, calibrating post-surgery muscle properties would have been an extremely challenging task, especially given that pelvic sarcoma patients experience significant anatomical changes. The personalized pre-surgery muscle properties were a much more accessible alternative. Static optimization that minimized the sum of squares of muscle activations was utilized to predict the muscle forces, which were later used to compute hip joint contact force. However, static optimization often underestimates muscle forces and thus joint contact forces as well (Kian et al., 2019). Despite this drawback, to the authors’ knowledge, no previous study used personalized hip joint contact forces for pelvis FE analyses. Most studies simply used published hip joint contact forces measured with an instrumented hip replacement (Bergmann et al., 2001) and re-expressed in the pelvis coordinate system (Soloviev et al., 2023; Zhu et al., 2023).

Third, the use of only two load cases from a single task may have also affected our results. Only two selected load cases during the gait cycle were used to predict the durability of the compression screws. As evident in various previous studies, other load cases in the gait cycle and load cases from other activities might be important to consider when predicting the likelihood of mechanical failures (Bergmann et al., 2010; Maslov et al., 2021; Zhu et al., 2023). However, the effect of loading conditions on predicted implant fixation durability is beyond the scope of the present study. Furthermore, muscle forces estimated by the neuromusculoskeletal model were not applied to our FE model. However, the influence of muscle forces on pelvis-implant FE simulations is not well understood. Some previous studies have argued that the presence of muscle forces in computational models has little influence on the simulated stress distribution in the pelvic implant, especially for the resected pelvis where a considerable amount of attached muscles were removed (Dalstra and Huiskes, 1995; Iqbal et al., 2017). A future study should investigate how analysis of multiple load cases from multiple movement conditions, including and excluding muscle forces, affects the predicted durability of custom pelvic implants.

Fourth, due to lack of available experimental data, values of various FE model parameters were taken from the literature. For example, the material properties of the 3D-printed implant were taken from a reputable study (Niinomi and Boehlert, 2015); the material properties of the remaining pelvic bone were calculated using established image processing techniques and strongly correlated empirical relations (Ebbesen et al., 1997; Anderson et al., 2005; Eggermont et al., 2019; Fleps et al., 2020; Babazadeh Naseri et al., 2021); all the coefficients of friction came from studies that conducted experimental measurements on orthopedic or dental implants (Guda et al., 2008; de Vries et al., 2022); the inferred pretension forces were determined through a series of steps involving estimates of various other parameters. The actual values of these parameters could be substantially different from the estimated values. As a more specific example, the actual pretension forces to which the screws were subject could be far from our estimated values, and the difference between actual and inferred pretension forces could alter FE predictions of the likelihood of screw failure. Although the FE simulation results presented in this study could not be validated experimentally or tested on a different implant design due to the patient-specific nature of the FE model, standard practices for pelvis FE analysis were followed.

Fifth, the present study examined the influence of compression screw modeling methods on predicted fixation durability of the entire postoperative pelvic assembly. The influence of these methods on the stress distribution in the bone was outside of the scope of the study. Due to the retrospective nature of the study, any variations in bone stress distributions caused by the different screw modeling methods could not be evaluated qualitatively, as the postoperative conditions of the bone were not available. Nonetheless, a better understanding of how bone screw modeling methods may influence the predicted stress state in the bone would be valuable to know, and a future study designed to investigate this issue should be conducted. Lastly, while computational modeling studies should ideally perform verification, validation, and uncertainty quantification (VVUQ) processes (ASME International Standard VVUQ 1-2022, 2022), we were unable to perform all three processes for the present study due to limitations in available experimental data and computational speed. For verification, we were able to compare the likelihood of screw pullout and fatigue failure predicted by the different screw modeling methods. The high similarity of the predictions generated by the three iterative physics-based methods and the non-iterative Abaqus BL-CL method provide confidence that these four modeling methods were implemented properly and satisfied the relevant physics relationships. For validation, we were able to evaluate model predictions of screw pullout and fatigue failure only qualitatively by comparing with clinical observations made in the subject being modeled, as *in vivo* quantitative measurement of bone screw stresses and strains is not currently possible. The three iterative physics-based methods and the non-iterative Abaqus BL-CL method all predicted that Cancellous 3 was susceptible to pullout failure, which was consistent with the clinical observation that Cancellous 3 indeed experienced a pullout failure. For uncertainty quantification, we were unable to evaluate the sensitivity of model predictions to uncertainties in model parameter values due to the significant amount of computational time (between 10 and 72 h) required for simulating each screw modeling method. Despite these limitations, this study was still able to demonstrate the advantages and disadvantages of various screw modeling methods used within pelvis-implant FE models for fixation durability analysis.

In conclusion, this study used a patient-specific hemipelvis with custom implant FE model to compare the likelihood of compression screw pullout and fatigue failure predicted by six distinct screw modeling methods. Our findings suggest that the choice of screw modeling method can dramatically alter FE-based predictions of pullout and fatigue failure likelihood for compression screws used in custom pelvic implants. The BL-CL modeling method, where the Abaqus build-in bolt load functionality was utilized, generated fast and reliable simulation results compared to other screw modeling methods. When this Abaqus built-in functionality is unavailable, the iterative physics-based FOR method was the best alternative. The study demonstrated the importance of utilizing a physiological screw modeling method for custom pelvic implant fixation durability studies involving compression screws. By incorporating an appropriate compression screw modeling method within patient-specific pelvis-custom implant FE models, this study paves the way for building better standards for evaluating custom implant designs *in silico* so that implant longevity and functionality can be maximized for each unique patient.

## Data Availability

The datasets presented in this study can be found in online repositories. The names of the repository/repositories and accession number(s) can be found below: The experimental walking data used to define bone loading for this study are available at https://simtk.org/projects/pelvic-screws.

## References

[B1] Alkan I. Sertgöz A. Ekici B. (2004). Influence of occlusal forces on stress distribution in preloaded dental implant screws. J. Prosthet. Dent. 91, 319–325. 10.1016/j.prosdent.2004.01.016 15116032

[B2] Anderson A. E. Peters C. L. Tuttle B. D. Weiss J. A. (2005). Subject-specific finite element model of the pelvis: development, validation and sensitivity studies. J. Biomech. Eng. 127, 364–373. 10.1115/1.1894148 16060343

[B3] Angelini A. Trovarelli G. Berizzi A. Pala E. Breda A. Ruggieri P. (2019). Three-dimension-printed custom-made prosthetic reconstructions: from revision surgery to oncologic reconstructions. Int. Orthop. (SICOT) 43, 123–132. 10.1007/s00264-018-4232-0 30467646

[B4] Ao D. Vega M. M. Shourijeh M. S. Patten C. Fregly B. J. (2022). EMG-driven musculoskeletal model calibration with estimation of unmeasured muscle excitations via synergy extrapolation. Front. Bioeng. Biotechnol. 10, 962959. (Accessed July 5, 2023). 10.3389/fbioe.2022.962959 36159690 PMC9490010

[B5] ASME International Standard VVUQ 1-2022 (2022). Verification, validation, and uncertainty quantification terminology in computational modeling and simulation. New York, NY: American Society of Mechanical Engineers.

[B6] Babazadeh Naseri A. Dunbar N. J. Baines A. J. Akin J. E. Higgs III C. F. Fregly B. J. (2021). Heterogeneous material mapping methods for patient-specific finite element models of pelvic trabecular bone: a convergence study. Med. Eng. and Phys. 96, 1–12. 10.1016/j.medengphy.2021.07.012 34565547

[B7] Balaji N. N. Chen W. Brake M. R. W. (2020). Traction-based multi-scale nonlinear dynamic modeling of bolted joints: formulation, application, and trends in micro-scale interface evolution. Mech. Syst. Signal Process. 139, 106615. 10.1016/j.ymssp.2020.106615

[B8] Bergmann G. Deuretzbacher G. Heller M. Graichen F. Rohlmann A. Strauss J. (2001). Hip contact forces and gait patterns from routine activities. J. Biomechanics 34, 859–871. 10.1016/S0021-9290(01)00040-9 11410170

[B9] Bergmann G. Graichen F. Rohlmann A. Bender A. Heinlein B. Duda G. N. (2010). Realistic loads for testing hip implants. Bio-Medical Mater. Eng. 20, 65–75. 10.3233/BME-2010-0616 20592444

[B10] Böhme J. Shim V. Höch A. Mütze M. Müller C. Josten C. (2012). Clinical implementation of finite element models in pelvic ring surgery for prediction of implant behavior: a case report. Clin. Biomech. 27, 872–878. 10.1016/j.clinbiomech.2012.06.009 22770881

[B11] Broekhuis D. Boyle R. Karunaratne S. Chua A. Stalley P. (2022). Custom designed and 3D-printed titanium pelvic implants for acetabular reconstruction after tumour resection. Hip Int. 33, 905–915. 10.1177/11207000221135068 36408844 PMC10486168

[B12] Budynas R. G. Nisbett J. K. (2011). in Shigley’s mechanical engineering design (New York: McGraw-Hill), 286–294.

[B13] Chapman J. R. Harrington R. M. Lee K. M. Anderson P. A. Tencer A. F. Kowalski D. (1996). Factors affecting the pullout strength of cancellous bone screws. J. Biomechanical Eng. 118, 391–398. 10.1115/1.2796022 8872262

[B14] Chen G. Muheremu A. Yang L. Wu X. He P. Fan H. (2020). Three-dimensional printed implant for reconstruction of pelvic bone after removal of giant chondrosarcoma: a case report. J. Int. Med. Res. 48, 030006052091727. 10.1177/0300060520917275 PMC7160782 32290744

[B15] Dalstra M. Huiskes R. (1995). Load transfer across the pelvic bone. J. Biomechanics 28, 715–724. 10.1016/0021-9290(94)00125-N 7601870

[B16] Dannaway J. Dabirrahmani D. Sonnabend D. Martin A. Appleyard R. (2015). An investigation into the frictional properties between bone and various orthopedic implant surfaces — implant stability. J. Musculoskelet. Res. 18, 1550015. 10.1142/S0218957715500153

[B17] Delp S. L. Anderson F. C. Arnold A. S. Loan P. Habib A. John C. T. (2007). OpenSim: open-source software to create and analyze dynamic simulations of movement. IEEE Trans. Biomed. Eng. 54, 1940–1950. 10.1109/TBME.2007.901024 18018689

[B18] Deutsches Institut für Normung (1991). DIN 946, Determination of coefficient of friction of bolt-nut assemblies under specified conditions.

[B19] de Vries E. Sánchez E. Janssen D. Matthews D. van der Heide E. (2022). Predicting friction at the bone – implant interface in cementless total knee arthroplasty. J. Mech. Behav. Biomed. Mater. 128, 105103. 10.1016/j.jmbbm.2022.105103 35121426

[B20] Dong E. Iqbal T. Fu J. Liu B. Guo Z. Cuadrado A. (2019). Preclinical strength checking for artificial pelvic prosthesis under multi-activities - a case study. J. Bionic Eng. 16, 1092–1102. 10.1007/s42235-019-0121-5

[B21] Dong E. Wang L. Iqbal T. Liu Y. He J. Zhao B. (2018). Finite element analysis of the pelvis after customized prosthesis reconstruction. J. Bionic Eng. 15, 443–451. 10.1007/s42235-018-0035-7

[B22] Ebbesen E. N. Thomsen J. S. Mosekilde Li. (1997). Nondestructive determination of iliac crest cancellous bone strength by pQCT. Bone 21, 535–540. 10.1016/S8756-3282(97)00196-8 9430244

[B23] Eggermont F. Verdonschot N. Linden Y. van der Tanck E. (2019). Calibration with or without phantom for fracture risk prediction in cancer patients with femoral bone metastases using CT-based finite element models. PLOS ONE 14, e0220564. 10.1371/journal.pone.0220564 31361790 PMC6667162

[B24] Fleps I. Bahaloo H. Zysset P. K. Ferguson S. J. Pálsson H. Helgason B. (2020). Empirical relationships between bone density and ultimate strength: a literature review. J. Mech. Behav. Biomed. Mater. 110, 103866. 10.1016/j.jmbbm.2020.103866 32957183

[B25] Fletcher J. W. A. Ehrhardt B. MacLeod A. Whitehouse M. R. Gill H. Preatoni E. (2019). Non-locking screw insertion: No benefit seen if tightness exceeds 80% of the maximum torque. Clin. Biomech. 70, 40–45. 10.1016/j.clinbiomech.2019.07.009 31386975

[B26] Guda T. Ross T. A. Lang L. A. Millwater H. R. (2008). Probabilistic analysis of preload in the abutment screw of a dental implant complex. J. Prosthet. Dent. 100, 183–193. 10.1016/S0022-3913(08)60177-8 18762030

[B27] Guo Z. Peng Y. Shen Q. Li J. He P. Yuan P. (2023). Reconstruction with 3D-printed prostheses after type I + II + III internal hemipelvectomy: finite element analysis and preliminary outcomes. Front. Bioeng. Biotechnol. 10, 1036882. 10.3389/fbioe.2022.1036882 36698627 PMC9868148

[B28] Hao Z. Wan C. Gao X. Ji T. (2011). The effect of boundary condition on the biomechanics of a human pelvic joint under an axial compressive load: a three-dimensional finite element model. J. Biomechanical Eng. 133, 101006. 10.1115/1.4005223 22070331

[B29] Inceoglu S. Akbay A. McLain R. F. (2006). Stress relaxation at the bone–pedicle screw interface in human bone. Spine 31, 1321–1326. 10.1097/01.brs.0000218478.70656.63 16721293

[B30] Iqbal T. Shi L. Wang L. Liu Y. Li D. Qin M. (2017). Development of finite element model for customized prostheses design for patient with pelvic bone tumor. Proc. Inst. Mech. Eng. H. 231, 525–533. 10.1177/0954411917692009 28639517

[B31] Ji T. Guo W. (2019). The evolution of pelvic endoprosthetic reconstruction after tumor resection. Ann. Jt. 4, 29. 10.21037/aoj.2019.06.01

[B32] Ji T. Yang Y. Tang X. Liang H. Yan T. Yang R. (2020). 3D-Printed modular hemipelvic endoprosthetic reconstruction following periacetabular tumor resection: early results of 80 consecutive cases. J. Bone Jt. Surg. 102, 1530–1541. 10.2106/JBJS.19.01437 32427766

[B33] Kian A. Pizzolato C. Halaki M. Ginn K. Lloyd D. Reed D. (2019). Static optimization underestimates antagonist muscle activity at the glenohumeral joint: a musculoskeletal modeling study. J. Biomech. 97, 109348. 10.1016/j.jbiomech.2019.109348 31668905

[B34] Lang L. A. Kang B. Wang R.-F. Lang B. R. (2003). Finite element analysis to determine implant preload. J. Prosthet. Dent. 90, 539–546. 10.1016/j.prosdent.2003.09.012 14668754

[B35] Lawson K. J. Brems J. (2001). Effect of insertion torque on bone screw pullout strength. Orthopedics 24, 451–454. 10.3928/0147-7447-20010501-12 11379993

[B36] Li G. Ao D. Vega M. M. Shourijeh M. S. Zandiyeh P. Chang S.-H. (2022). A computational method for estimating trunk muscle activations during gait using lower extremity muscle synergies. Front. Bioeng. Biotechnol. 10, 964359. 10.3389/fbioe.2022.964359 36582837 PMC9792665

[B37] Li G. Ao D. Vega M. M. Zandiyeh P. Chang S.-H. Penny A. N. (2024). Changes in walking function and neural control following pelvic cancer surgery with reconstruction. Front. Bioeng. Biotechnol. 12, 1389031. 10.3389/fbioe.2024.1389031 38827035 PMC11140731

[B38] Li Z. Alonso J. E. Kim J.-E. Davidson J. S. Etheridge B. S. Eberhardt A. W. (2006). Three-dimensional finite element models of the human pubic symphysis with viscohyperelastic soft tissues. Ann. Biomed. Eng. 34, 1452–1462. 10.1007/s10439-006-9145-1 16897423

[B39] Liang H. Ji T. Zhang Y. Wang Y. Guo W. (2017). Reconstruction with 3D-printed pelvic endoprostheses after resection of a pelvic tumour. Bone and Jt. J. 99-B, 267–275. 10.1302/0301-620X.99B2.BJJ-2016-0654.R1 28148672

[B40] Liu F. Fisher J. Jin Z. (2012). Computational modelling of polyethylene wear and creep in total hip joint replacements: effect of the bearing clearance and diameter. Proc. Institution Mech. Eng. Part J J. Eng. Tribol. 226, 552–563. 10.1177/1350650112441908

[B41] Marin J. (1962). in Mechanical behavior of engineering materials (Englewood Cliffs. N.J.: Prentice-Hall), 224.

[B42] Maslov L. Borovkov A. Maslova I. Soloviev D. Zhmaylo M. Tarasenko F. (2021). Finite element analysis of customized acetabular implant and bone after pelvic tumour resection throughout the gait cycle. Materials 14, 7066. 10.3390/ma14227066 34832464 PMC8618128

[B43] Morgan E. F. Unnikrisnan G. U. Hussein A. I. (2018). Bone mechanical properties in healthy and diseased states. Annu. Rev. Biomed. Eng. 20, 119–143. 10.1146/annurev-bioeng-062117-121139 29865872 PMC6053074

[B44] Niinomi M. (2008). Mechanical biocompatibilities of titanium alloys for biomedical applications. J. Mech. Behav. Biomed. Mater. 1, 30–42. 10.1016/j.jmbbm.2007.07.001 19627769

[B45] Niinomi M. Boehlert C. J. (2015). “Titanium alloys for biomedical applications,” in Advances in metallic biomaterials: tissues, materials and biological reactions. Editors Niinomi M. Narushima T. Nakai M. (Berlin, Heidelberg: Springer), 179–213. 10.1007/978-3-662-46836-4_8

[B46] Ozaki T. Hoffmann C. Hillmann A. Gosheger G. Lindner N. Winkelmann W. (2002). Implantation of hemipelvic prosthesis after resection of sarcoma. Clin. Orthop. Relat. Research® 396, 197–205. 10.1097/00003086-200203000-00030 11859244

[B47] Park D. W. Lim A. Park J. W. Lim K. M. Kang H. G. (2019). Biomechanical evaluation of a new fixation type in 3D-printed periacetabular implants using a finite element simulation. Appl. Sci. 9, 820. 10.3390/app9050820

[B48] Phillips A. T. M. Pankaj P. Howie C. R. Usmani A. S. Simpson A. H. R. W. (2007). Finite element modelling of the pelvis: inclusion of muscular and ligamentous boundary conditions. Med. Eng. and Phys. 29, 739–748. 10.1016/j.medengphy.2006.08.010 17035063

[B49] Rajagopal A. Dembia C. L. DeMers M. S. Delp D. D. Hicks J. L. Delp S. L. (2016). Full-body musculoskeletal model for muscle-driven simulation of human gait. IEEE Trans. Biomed. Eng. 63, 2068–2079. 10.1109/TBME.2016.2586891 27392337 PMC5507211

[B50] Sanyal A. Gupta A. Bayraktar H. H. Kwon R. Y. Keaveny T. M. (2012). Shear strength behavior of human trabecular bone. J. Biomech. 45, 2513–2519. 10.1016/j.jbiomech.2012.07.023 22884967 PMC3462285

[B51] Satpathy M. Jose R. M. Duan Y. Griggs J. A. (2022). Effects of abutment screw preload and preload simulation techniques on dental implant lifetime. JADA Found. Sci. 1, 100010. 10.1016/j.jfscie.2022.100010 36704641 PMC9873498

[B52] Seth A. Hicks J. L. Uchida T. K. Habib A. Dembia C. L. Dunne J. J. (2018). OpenSim: simulating musculoskeletal dynamics and neuromuscular control to study human and animal movement. PLOS Comput. Biol. 14, e1006223. 10.1371/journal.pcbi.1006223 30048444 PMC6061994

[B53] Shao Q. D. Yan X. Sun J. Y. Xu T. M. (2015). Internal hemipelvectomy with reconstruction for primary pelvic neoplasm: a systematic review. ANZ J. Surg. 85, 553–560. 10.1111/ans.12895 25387591

[B54] Shoberg R. S. (2000). Engineering fundamentals of threaded fastener design and analysis. Available at: https://hexagon.de/rs/engineering%20fundamentals.pdf.

[B55] Soloviev D. Maslov L. Zhmaylo M. (2023). Acetabular implant finite element simulation with customised estimate of bone properties. Materials 16, 398. 10.3390/ma16010398 36614737 PMC9822217

[B56] Sun W. Li J. Li Q. Li G. Cai Z. (2011). Clinical effectiveness of hemipelvic reconstruction using computer-aided custom-made prostheses after resection of malignant pelvic tumors. J. Arthroplasty 26, 1508–1513. 10.1016/j.arth.2011.02.018 21477973

[B57] Wang B. Sun P. Xie X. Wu W. Tu J. Ouyang J. (2015). A novel combined hemipelvic endoprosthesis for peri-acetabular tumours involving sacroiliac joint: a finite element study. Int. Orthop. (SICOT) 39, 2253–2259. 10.1007/s00264-015-2891-7 26183143

[B58] Wang J. Min L. Lu M. Zhang Y. Wang Y. Luo Y. (2019). Three-dimensional-printed custom-made hemipelvic endoprosthesis for primary malignancies involving acetabulum: the design solution and surgical techniques. J. Orthop. Surg. Res. 14, 389. 10.1186/s13018-019-1455-8 31775805 PMC6882053

[B59] Wang M. Liu T. Xu C. Liu C. Li B. Lian Q. (2022). 3D-printed hemipelvic prosthesis combined with a dual mobility bearing in patients with primary malignant neoplasm involving the acetabulum: clinical outcomes and finite element analysis. BMC Surg. 22, 357. 10.1186/s12893-022-01804-8 36203147 PMC9541076

[B60] Watson P. J. Dostanpor A. Fagan M. J. Dobson C. A. (2017). The effect of boundary constraints on finite element modelling of the human pelvis. Med. Eng. and Phys. 43, 48–57. 10.1016/j.medengphy.2017.02.001 28259612

[B61] Xu S. Guo Z. Shen Q. Peng Y. Li J. Li S. (2022). Reconstruction of tumor-induced pelvic defects with customized, three-dimensional printed prostheses. Front. Oncol. 12, 935059. 10.3389/fonc.2022.935059 35847863 PMC9282862

[B62] Yushkevich P. A. Gerig G. (2017). ITK-SNAP: an intractive medical image segmentation tool to meet the need for expert-guided segmentation of complex medical images. IEEE Pulse 8, 54–57. 10.1109/MPUL.2017.2701493 28715317

[B63] Zhou R. Xue H. Wang J. Wang X. Wang Y. Zhang A. (2022). Improving the stability of a hemipelvic prosthesis based on bone mineral density screw channel and prosthesis optimization design. Front. Bioeng. Biotechnol. 10, 892385. 10.3389/fbioe.2022.892385 35706507 PMC9189365

[B64] Zhou Y. Min L. Liu Y. Shi R. Zhang W. Zhang H. (2013). Finite element analysis of the pelvis after modular hemipelvic endoprosthesis reconstruction. Int. Orthop. 37, 653–658. 10.1007/s00264-012-1756-6 23318936 PMC3609974

[B65] Zhu Y. Babazadeh-Naseri A. Dunbar N. J. Brake M. R. W. Zandiyeh P. Li G. (2023). Finite element analysis of screw fixation durability under multiple boundary and loading conditions for a custom pelvic implant. Med. Eng. and Phys. 111, 103930. 10.1016/j.medengphy.2022.103930 36792235

