# Supplementary material for: Evaluation of finite element modeling methods for predicting compression screw failure in a custom pelvic implant
Source: Front Bioeng Biotechnol. 2024 Aug 20;12:1420870. doi: 10.3389/fbioe.2024.1420870 (PMC11372789; doi:10.3389/fbioe.2024.1420870)
Supplement: Supplementary file 1 [file DataSheet1.PDF]

Supplementary Material

Evaluation of Finite Element Modeling Methods for Predicting Compression Screw Failure in a Custom Pelvic Implant

1 Single-screw Model and Mesh Sensitivity Study

1.1 Methods

A single-screw model isolating the screw from other complex geometries in the hemipelvis model was developed in Abaqus to generate initial guesses for the iterative screw modeling methods. The single-screw model represented a typical trabecular screw and consisted of the screw, a cubic block representing trabecular bone, and a solid block representing the implant. The screw was inserted perpendicular to the bone-implant interface through a planned screw hole within the implant block. The far end surface of the bone block was fixed and prevented from moving in any translational directions.

This single-screw model was iterated to approximate the temperature difference or magnitude of compressive forces required to induce the inferred pretension forces. The initial guess of the single-screw model assumed the strain in the screw shank was equal to the induced thermal or mechanical strain in the screw free zone. For instance, the initial guess of the temperature change (ΔT) in each screw was calculated using $\Delta T = \frac{\varepsilon}{\alpha} = \frac{F_P}{A_{shank} E \alpha}$, where ε was the thermal strain, α was the coefficient of thermal expansion of the screw material (*i.e.*, Ti64), A_{shank} was the cross-section area of the screw shank, and E was the Young's Modulus; the initial guess of the magnitude of compressive forces (F_C) was obtained assuming $F_C = \frac{F_P A_{shank}}{A_{core}}$, where A_{core} was the cross-section area of the screw core. The initial guess was linearly interpolated until the axial force with the free zone of the single screw was within ±1 N difference from the inferred pretension forces. The magnitude of the temperature change or the compressive forces that produced the inferred pretension force with the free zone for each screw in the single-screw model was then used as the initial guess of the iterative process to induce the inferred pretension force in the hemipelvis model.

Using the single-screw model, we also conducted a mesh sensitivity analysis to assess the influence of mesh refinement and screw model selection on stress concentrations. The objective was to determine the distance required away from the screw shank where the simulated stresses remain undisturbed by stress concentration at the beginning of the screw core for the iterative models. Three levels of mesh refinement were investigated. The first level, denoted as "coarse" mesh, employed an element size of approximately 1 mm, which closely matched the element size of nine screws in the hemipelvis model (~0.8 mm) (Supplementary Figure 1). The subsequent refinement steps, labeled as "medium" and "fine" mesh, yielded element sizes of 0.5 mm and 0.25 mm, respectively. For the mesh sensitivity analysis, we imposed a 500 N pretension force on the screw free zone through the aforementioned single-screw iterative process. The von Mises stress (σ_{vM}) distribution on the screw surface was extracted from the model and processed for a comprehensive analysis of stress distribution in the screw system.

1.2 Results

All modeling methods and mesh sizes used in the mesh sensitivity analysis displayed stress peaks at the beginning of the screw core (Supplementary Figure 2), similar to the axial force peaks observed in the hemipelvis FE model during the pretension step. The peaks were partially attributable to stress concentration caused by the sharp geometric transition from the screw shank to the core. Notably, variations in stress values were observed at the region adjacent to the screw shank among models with different mesh sizes. This indicated that the stress predictions in this region were sensitive to mesh size. However, as the distance from the beginning of the screw core increased, the stress profiles converged to a stable plateau over the screw free zone. The distance required for a reliable evaluation of screw stresses, unaffected by stress concentration, varied significantly depending on the screw model to induce the inferred pretension force and mesh size. ISO exhibited adverse effects on stress distribution at the beginning of the screw core adjacent to the screw shank, extending up to one core diameter (*i.e.,* 3 mm). Interestingly, further mesh refinement did not significantly alleviate the stress concentration effects caused by the ISO model. On the contrary, when inducing the same pretension force, both FOR and ORT models demonstrated a shorter length of the screw free zone influenced by stress concentrations. The stress profiles indicated that the ORT model resulted in convergence within two elements away from the beginning of the screw core, while the FOR model achieved convergence within one element. Additionally, the ORT model exhibited a smaller stress concentration at the beginning of the threaded screw compared to the FOR model. However, the ORT model required a slightly larger distance (one more layer of elements) to attain reliable stress evaluations than the FOR model. Consequently, the FOR model showcased superior performance in achieving reliable stress evaluations within a shorter distance than other screw pretension application methods.

2 Supplementary Figures

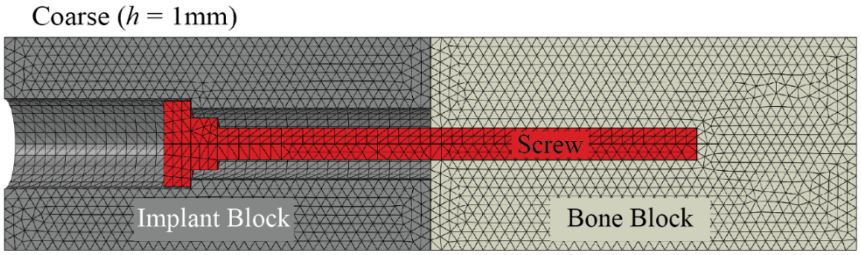

Supplementary Figure 1. The single screw model meshed with coarse elements. The model was used for mesh sensitivity analysis to understand the relations between the element size and stress concentration.

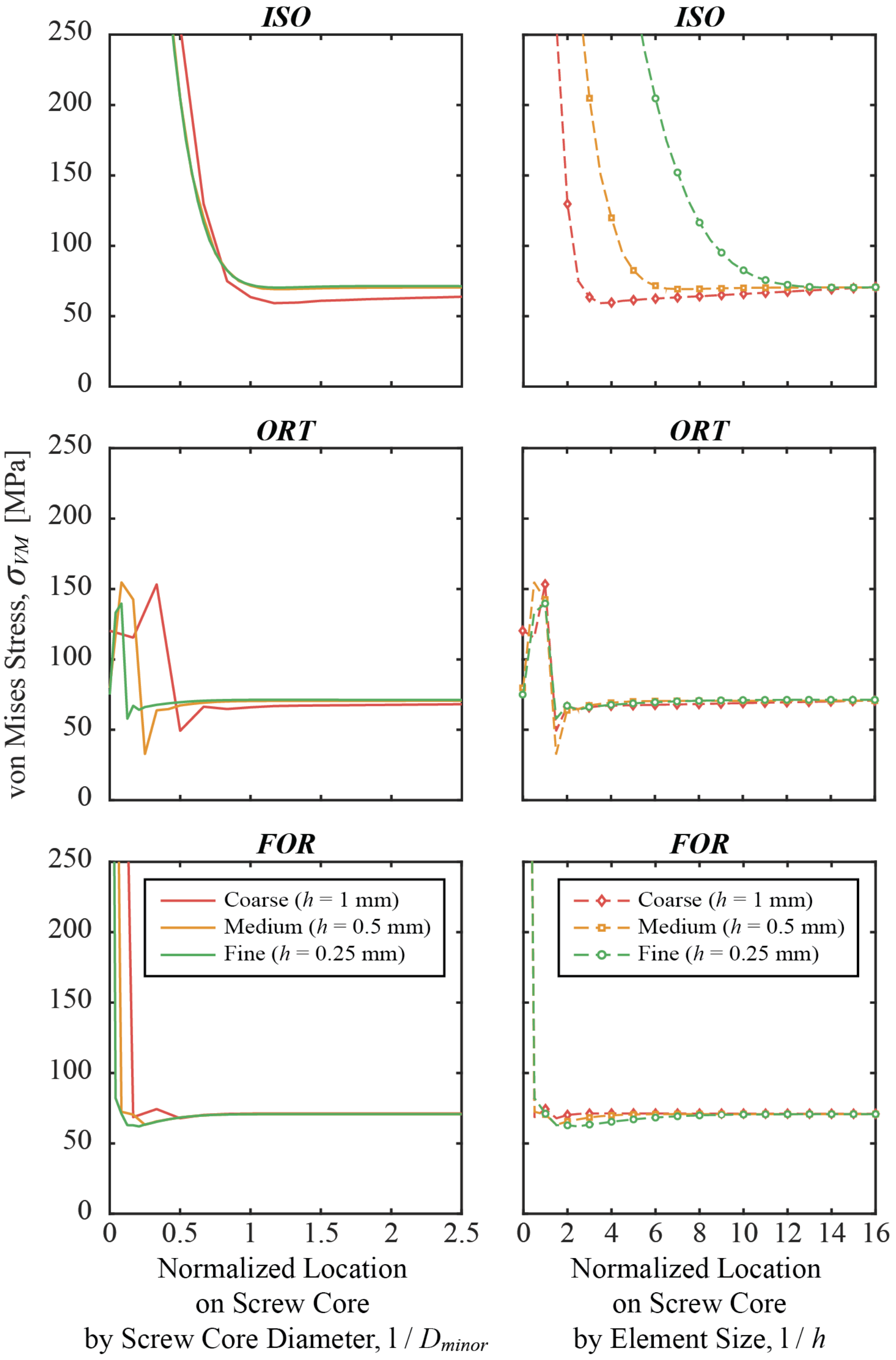

Supplementary Figure 2. The von Mises stress alone the screw core in the single screw model. The location on the screw core was normalized by the screw core diameter for the plots in the left column or by the element size for the plots in the right column.
